# Multiple phenotypic traits as triggers of host attacks towards ant symbionts: body size, morphological gestalt, and chemical mimicry accuracy

**DOI:** 10.1186/s12983-021-00427-8

**Published:** 2021-09-19

**Authors:** Christoph von Beeren, Adrian Brückner, Philipp O. Hoenle, Bryan Ospina-Jara, Daniel J. C. Kronauer, Nico Blüthgen

**Affiliations:** 1 Ecological Networks, Department of Biology, Technical University of Darmstadt, Darmstadt, Germany; 2 Division of Biology and Biological Engineering, California Institute of Technology, Pasadena, USA; 3 Department of Biology, University of Valle, Cali, Colombia; 4 Laboratory of Social Evolution and Behavior, The Rockefeller University, New York City, USA

**Keywords:** *Eciton*, Ant guest, Chemical mimicry, Myrmecophile, Body size, Morphological gestalt, Cuticular hydrocarbons, Symbiont community, Social parasite

## Abstract

**Background:**

Ant colonies are plagued by a diversity of arthropod guests, which adopt various strategies to avoid or to withstand host attacks. Chemical mimicry of host recognition cues is, for example, a common integration strategy of ant guests. The morphological gestalt and body size of ant guests have long been argued to also affect host hostility, but quantitative studies testing these predictions are largely missing. We here evaluated three guest traits as triggers of host aggression—body size, morphological gestalt, and accuracy in chemical mimicry—in a community of six *Eciton* army ant species and 29 guest species. We quantified ant aggression towards 314 guests in behavioral assays and, for the same individuals, determined their body size and their accuracy in mimicking ant cuticular hydrocarbon (CHC) profiles. We classified guests into the following gestalts: protective, myrmecoid, staphylinid-like, phorid-like, and larval-shaped. We expected that (1) guests with lower CHC mimicry accuracy are more frequently attacked; (2) larger guests are more frequently attacked; (3) guests of different morphological gestalt receive differing host aggression levels.

**Results:**

Army ant species had distinct CHC profiles and accuracy of mimicking these profiles was variable among guests, with many species showing high mimicry accuracy. Unexpectedly, we did not find a clear relationship between chemical host similarity and host aggression, suggesting that other symbiont traits need to be considered. We detected a relationship between the guests’ body size and the received host aggression, in that diminutive forms were rarely attacked. Our data also indicated that morphological gestalt might be a valuable predictor of host aggression. While most ant-guest encounters remained peaceful, host behavior still differed towards guests in that ant aggression was primarily directed towards those guests possessing a protective or a staphylinid-like gestalt.

**Conclusion:**

We demonstrate that CHC mimicry accuracy does not necessarily predict host aggression towards ant symbionts. Exploitation mechanisms are diverse, and we conclude that, besides chemical mimicry, other factors such as the guests’ morphological gestalt and especially their body size might be important, yet underrated traits shaping the level of host hostility against social insect symbionts.

**Supplementary Information:**

The online version contains supplementary material available at 10.1186/s12983-021-00427-8.

## Background

The colonies of social insects are plagued by numerous predators, parasitoids, parasites, and commensals [1–4]. Among these are organisms as diverse as chimpanzees and anteaters preying on social insects [5–8], endoparasitic nematodes and fungi altering the behavior of social insect workers [3, 9–12], and various arthropods taking advantage of the abundant resources accumulating in and around social insect colonies [13–16]. The latter are particularly species-rich, containing hundreds of thousands of species from diverse arthropod taxa such as mites, spiders, flies, beetles, wasps, millipedes, isopods, silverfish, and crickets [13, 14, 16–18].

Living in close proximity to or even within social insect nests provides several benefits to arthropod guests [2, 4, 13, 14]. Among others, these guests are supposedly protected from their own predators, parasites, and parasitoids [4]. Further, they exploit the colony’s food resources or feed directly on the social insects [4, 16, 19]. In order to achieve this intimacy, social insect guests usually possess a multitude of countermeasures against colony defenses [2, 4, 14, 16, 18, 20–25], including mimicry of chemical host recognition cues or chemical hiding [25–32]. Social insects recognize colony members and distinguish them from intruders to a great extent via cuticular hydrocarbons (CHCs) [33–35]. Mimicking these olfactory cues—or expressing no detectable olfactory cues at all—are strategies expected to hamper the recognition of guests as colony intruders, thus facilitating successful exploitation of social insect societies [24, 30, 34, 36]. Besides chemical mimicry and chemical hiding (sensu [32]), however, behavioral adaptations, vibroacoustic mimicry, chemical trickery (e.g., adoption glands [37]) and chemical weaponry (e.g., defensive glands [21]), as well as the morphological gestalt and body size of social insect guests might be equally important for successful exploitation of host colonies [23, 27, 37–41].

**Fig. 1:**
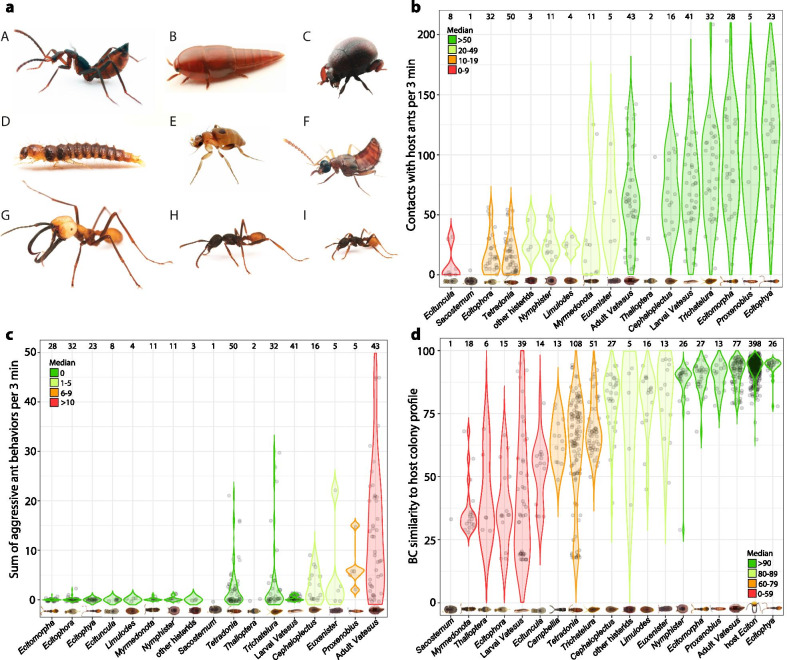
Morphological gestalt, ant behaviors towards ecitophiles, and ecitophile mimicry accuracy. **a**: (A)* Ecitophya* rove beetle representing the myrmecoid gestalt, (B)* Vatesus* rove beetle as well as (C)* Nymphister* histerid beetle the protective gestalt, (D)* Vatesus* rove beetle larva the larval-shaped gestalt, (E) *Ecitophora* phorid fly the phorid-like gestalt, and (F) *Tetradonia* rove beetle the staphylinid-like gestalt. *Eciton burchellii* workers representing different army ant size classes: (G) major, (H) intermediate, and (I) minor. Violin jitter plots visualizing **b** the number of host contacts, **c** the sum of aggressive ant encounters (*chasing*, *snapping*, *stinging,* and *seizing*), and **d** CHC profile similarities to the colonies’ average worker profile (BC similarity). For better data visualization, species within genera are combined. Additional files 1 and 2 include information at the species level. Sample sizes are given above violin plots, which are ordered according to the groups’ medians. The category 'other histerids' includes the species *Aphanister* sp. 1, *Cheilister* cf. *lucidulus*, *Psalidister furcatus*, and *Sternocoelopsis* cf. *nevermanni*. Images are not to scale. Image credits: Daniel Kronauer

Many social insect guests are generalized in their appearance and differ little from their free-living relatives, while others possess highly modified morphologies [2, 4, 13, 14, 19, 42]. It has long been argued that these morphological modifications represent adaptations to social insect exploitation [42–46], in particular because similar and functionally equivalent body shapes exist among distantly related guests (e.g., [2, 4, 13, 14, 42]). The convergent/parallel evolution of an ant-like, or myrmecoid body shape in some army ant-associated rove beetles is one of the most fascinating examples of an adaptive morphological gestalt [42, 44, 47] (Fig. 1a-A). Myrmecoids have a narrowed waist with an expanded abdomen (i.e., a petiolate abdomen; [47]), geniculate antennae, and long legs relative to their body size, often resembling their host ants to a remarkable degree [14, 42, 48]. They live inside the army ants’ temporary nests (bivouacs) and therefore frequently come into physical contact with host workers [19, 38, 49]. Instead of being attacked and expelled, host workers ignore or even groom these cleptoparasitic guests [19, 38]. Apparently, myrmecoid rove beetles have cracked the ants’ communication system as they are treated like members of the colony [19, 38, 50].

Instead of mimicking host ants, other social insect guests possess a protective morphology [2, 4, 13], defensive anatomical modifications that already existed in ant and termite guests at the very onset of social insect evolution in the Mesozoic [51–53]. For instance, the drop-shaped gestalt, often referred to as limuloid (referring to the horse-shoe crab genus *Limulus*), is a common type of protective morphology encountered in diverse and unrelated taxa of social insect guests [2, 13, 54] (Fig. 1a-B). Limuloids possess laterally expanded body plates that form a protective shield for the head and most appendages [2, 43, 51]. These guests are difficult to catch for social insects due to their swift movements and agility, accompanied by a body shape that easily slips through mandibles during attacks [2, 14, 26, 43, 55]. Morphological protection is also the hallmark of histerid beetles, common guests in social insect nests [14, 56, 57]. With their robust, heavily sclerotized body with retractable appendages [56, 58], these slowly moving beetles are the functional equivalent of tortoises in the insect world (Fig. 1a-C). When fully retracted, no points of attack remain for the ants, leaving the beetles usually unharmed during aggressive encounters [14, 45, 53, 59, 60].

In his seminal review about social insect symbionts, Kistner [2] drew a connection between morphological gestalt and the degree of social integration into the ant society. For instance, he argued that limuloid guests are usually aggressed by approaching social insect workers, while myrmecoid guests are treated peacefully instead [2]. Other than these basic observations by Kistner and some others (e.g., [2, 14, 20, 27, 42, 43, 61]), quantitative studies assessing morphological gestalt as a predictor variable of host aggression against social insect guests are missing.

Besides their morphological gestalt, another characteristic of social insect guests is their relatively small size, which rarely exceeds the size of their host workers [14]. Body size relationships play a central role in structuring various types of ecological interactions [62], including food webs [63–67]. Several authors have suggested that body size also affects the interactions between social insects and their guests [2, 4, 13, 14, 43, 68, 69]. Parmentier et al. recently provided the first quantitative evidence for this relationship in wood ant-associated guests ([70, 71]; but see also [72]). In fact, most social insect guests appear to be smaller than their hosts and diminutive body size is common [2, 4, 13, 14], likely impeding recognition and thus protecting the intruders from attacks by host workers [2, 14, 73]. However, body size has not been studied intensively as a predictor of host attacks [14, 70–72], and it remains largely unclear whether this trait is indeed related to host tolerance.

The astounding diversity of social insect guests associated with colonies of Neotropical army ants [15, 20, 49, 74, 75] provides the opportunity to synergistically investigate multiple phenotypic traits that presumably facilitate host exploitation. We recently showed that 62 species of ant guests, or myrmecophiles, from diverse taxa infiltrate the colonies of *Eciton* army ants in a Costa Rican population [75]. Among these were species with different morphological gestalt, as well as diminutive and large-bodied guests [75]. Using standardized behavioral tests in laboratory nests, we studied the effect of three guest traits on the degree of host aggression towards *Eciton* myrmecophiles (also called ecitophiles): accuracy in chemical mimicry of host recognition cues, body size, and morphological gestalt. We tested the following three hypotheses: (a) ecitophiles with lower chemical mimicry accuracy of host profiles are more frequently attacked; (b) larger ecitophiles are more frequently attacked; (c) ecitophiles of different morphological gestalt receive differing levels of host attacks.

## Methods

### Specimen collection and research permit

We collected ecitophiles during nocturnal colony emigrations at La Selva Biological Station (LSBS)—a lowland tropical rainforest in Costa Rica (GPS data: 10° 25′ 19.2″ N, 84° 0′ 54″ W; 35 m–137 m a.s.l.). Ecitophiles participating in host colony emigrations are considered to be obligate symbionts of *Eciton* army ants ([20]; but see also [75] for possible exceptions). Details on collection methods, specimen deposition, and species identifications were published previously [75]. Army ants and ecitophiles were collected during one field trip to Costa Rica from February 2017 to April 2017. Ecitophiles of the following host species were studied: *Eciton burchellii* (Westwood, 1842) (subspecies *E. burchellii foreli* Mayr, 1886), *E. dulcium* Forel, 1912, *E. hamatum* (Fabricius, 1782), *E. lucanoides* Emery, 1894, *E. mexicanum* Roger, 1863, and *E. vagans* (Olivier, 1792) (Table 1). Army ant colony identification codes (IDs) and sample IDs (Additional file 1) correspond to IDs used in previous publications [38, 58, 59, 75–77]. Photo-stacked images of all herein studied species have been published earlier [75] and are accessible on the website of the Barcode of Life Data System (http://www.boldsystems.org/). Collection permits, export permits and research permits were issued by the ‘Ministry of the Environment, Energy and Technology’ and the ‘National Commission for Biodiversity Management’ (MINAET; permit number: R- 007–2017-OT-CONAGEBIO).

**Table 1:** Overview of sample sizes

Army ant species	Behavioral tests		Analysis of CHC profiles		
	No. of colonies	No. of ecitophiles	No. of colonies	No. of workers	No. of ecitophiles
*Eciton burchellii*	2*	114	3*	82	148
*Eciton dulcium*	2	43	3	81	47
*Eciton hamatum*	1	48	2	63	93
*Eciton lucanoides*	1	32	2	52	105
*Eciton mexicanum*	1	34	2	60	65
*Eciton vagans*	2	43	2	60	42

Given are the number of colonies and the number of specimens used for behavioural tests and analyses of CHC profiles per army ant species

*Including the *E. burchellii* colony EB15 studied in 2014 (see methods, [38])

### Army ant behavior towards ecitophiles

We studied the ants’ behavior towards ecitophiles in laboratory settings at the field site. We examined ant behavior towards 314 ecitophiles belonging to 29 species (Table 2). These included twelve rove beetle species, six histerid beetle species, three ptiliid beetle species, one water scavenger beetle species, six phorid fly species, and one silverfish species (Table 2; Additional file 1). Ecitophiles were exclusively tested with workers from their colony of origin. In total, behavioral tests were conducted in eight army ant colony fragments: one *E. burchellii* colony, two *E. dulcium* colonies, one *E. hamatum* colony, one *E. lucanoides* colony, one *E. mexicanum* colony, and two *E. vagans* colonies (Table 1).

**Table 2:** Overview of phenotypic traits per ecitophile species

Order	Family	Ecitophile species	Morph. gestalt	Host specificity	Dry weight ± SD	Contacts ± SD	AI ± SD	BCS ± SD
Coleoptera	Histeridae	*Aphanister* sp.1	Protective	Unknown	0.43 (1)	21 (1)	0.00	61 (1)
		*Cheilister* cf. *lucidus*	Protective	Unknown	0.48 (1)	23 (1)	0.00	39 (1)
		*Psalidister furcatus*	Protective	Unknown	0.75 (1)	46 (1)	0	83 (1)
		*Euxenister caroli*	Protective	Specialist	3.84 ± 1.45 (4)	63 ± 35 (4)	0.15 ± 0.23	85 ± 7 (4)
		*Euxenister wheeleri*	Protective	Specialist	2.86 ± 0.23 (9)	29 (1)	0.07	80 ± 18 (9)
		*Nymphister kronaueri*	Protective	Specialist	0.74 ± 0.13 (26)	27 ± 12 (11)	0.01 ± 0.01	86 ± 13 (26)
		*Sternocoelopsis* cf. *nevermanni*	Protective	Unknown	1.06 ± 0.04 (2)	–	–	92 ± 1 (2)
	Hydrophylidae	*Sacosternum* aff. *lebbinorum*	Protective	Unknown	0.31 (1)	4 (1)	0	33 (1)
	Ptiliidae	*Cephaloplectus mus*	Protective	Generalist	0.56 ± 0.12 (26)	–	–	79 ± 12 (27)
		*Limulodes* sp. 2	Protective	Specialist	0.04 ± 0.02 (2)	25 ± 1 (2)	0.00 ± 0.00	–
		*Limulodes* sp. 3	Protective	Moderate specificity	0.05 ± 0.01 (11)	–	–	84 ± 7 (11)
	Staphylinidae	*Campbellia lucanoides*	Myrmecoid	Specialist	0.47 ± 0.07 (11)	–	–	64 ± 9 (13)
		*Ecitopora* sp. 2	Staphylinid-like	Moderate specificity	0.391 (1)	–	–	59 (1)
		*Ecitomorpha* cf. *breviceps*	Myrmecoid	Specialist	0.44 ± 0.04 (3)	82 ± 42 (4)	0.01 ± 0.01	88 ± 6 (4)
		*Ecitomorpha* cf. *nevermanni*	Myrmecoid	Specialist	0.67 ± 0.15 (22)	95 ± 43 (24)	0.00 ± 0.00	90 ± 6 (23)
		*Ecitophya gracillima*	Myrmecoid	Specialist	–	–	–	89 ± 9 (3)
		*Ecitophya simulans*	Myrmecoid	Specialist	1.33 ± 0.51 (23)	115 ± 43 (23)	0.00 ± 0.00	94 ± 2 (22)
		*Myrmedonota* sp. 1	Staphylinid-like	Specialist	0.15 ± 0.03 (25)	36 ± 46 (11)	0.00 ± 0.01	37 ± 11 (18)
		*Proxenobius borgmeieri*	Staphylinid-like	Specialist	4.46 ± 0.54 (13)	97 ± 42 (5)	0.11 ± 0.13	89 ± 5 (13)
		*Tetradonia* cf. *marginalis*	Staphylinid-like	Moderate specificity	0.57 ± 0.07 (32)	12 ± 13 (19)	0.05 ± 0.09	74 ± 8 (32)
		*Tetradonia laselvensis*	Staphylinid-like	Moderate specificity	0.66 ± 0.08 (12)	18 ± 17 (10)	0.01 ± 0.02	62 ± 13 (12)
		*Tetradonia laticeps*	Staphylinid-like	Generalist	0.48 ± 0.09 (29)	23 ± 14 (12)	0.23 ± 0.22	38 ± 19 (29)
		*Tetradonia lizonae*	Staphylinid-like	Specialist	0.56 ± 0.10 (23)	15 ± 18 (9)	0.16 ± 0.15	70 ± 16 (24)
		*Tetradonia tikalensis*	Staphylinid-like	Moderate specificity	0.32 (1)	–	–	63 (1)
		*Vatesus* aff. *goianus*	Ad: Protective La: larval-shaped	Specialist	Ad: 2.35 ± 0.45 (34) La: 1.16 ± 0.48 (7)	Ad: 42 ± 22 (14) La 74 ± 24 (7)	Ad: 0.19 ± 0.13 0.01 ± 0.01	Ad: 90 ± 7 (33) La: 29 ± 8 (5)
		*Vatesus* cf. *clypeatus* sp. 1	Ad: Protective La: larval-shaped	Specialist	Ad: 4.69 ± 0.46 (6) La: 2.23 ± 1.12 (14)	Ad: 123 ± 15 (7) La: 56 ± 26 (14)	Ad: 0.15 ± 0.04 La: 0.00 ± 0.01	Ad: 89 ± 2 (7) La: 30 ± 16 (14)
		*Vatesus* cf. *clypeatus* sp. 2	Ad: Protective La: larval-shaped	Moderate specificity	Ad: 4.39 ± 0.64 (23) La: 1.76 ± 0.64 (31)	Ad: 65 ± 35 (21) La: 79 ± 43 (21)	Ad: 0.19 ± 0.15 La: 0.01 ± 0.03	Ad: 91 ± 6 (24) La: 54 ± 26 (23)
		*Vatesus* cf. *clypeatus* sp. 3	Ad: Protective	Specialist	Ad: 4.01 ± 0.33 (5)	–	–	Ad: 93 ± 1 (5)
Diptera	Phoridae	*Ecitophora bruchi*	Phorid-like	Specialist	0.03 ± 0.01 (6)	14 ± 7 (6)	0.00 ± 0.00	–
		*Ecitophora* cf. *comes* sp. 1	Phorid-like	Generalist	0.06 ± 0.02 (10)	16 ± 18 (9)	0.05 ± 0.13	32 ± 7 (5)
		*Ecitophora pilosula*	Phorid-like	Specialist	0.09 ± 0.02 (4)	22 ± 20 (10)	0.00 ± 0.00	44 ± 25 (5)
		*Ecitophora varians*	Phorid-like	Specialist	0.05 ± 0.02 (5)	7 ± 7 (5)	0.00 ± 0.00	40 ± 9 (5)
		*Ecituncula tarsalis*	Phorid-like	Moderate specificity	0.03 ± 0.01 (20)	9 ± 14 (8)	0.00 ± 0.00	52 ± 13 (14)
		*Thalloptera fuscipalpis*	Phorid-like	Moderate specificity	0.10 ± 0.04 (5)	64 ± 48 (2)	0.00 ± 0.00	45 ± 18 (5)
Thysanura	Nicoletiidae	*Trichatelura manni*	Protective	Generalist	2.39 ± 1.14 (48)	85 ± 39 (32)	0.07 ± 0.12	69 ± 11 (48)

Definitions of morphological gestalts are given in the methods. Host specificities of ecitophiles were evaluated in a separate study [75]. Host specificity categories (specialist, moderate, generalist, unknown) were defined according to the metric *d’* (Kullback–Leibler index): range of *d’* between 0.00–0.10 = generalist, 0.11–0.25 = moderate specificity, 0.26–0.52 = host specialist. Host specificities of rare species (< 5 specimens collected; see [75]) were defined as 'unknown'. Body size was calculated as ecitophiles’ dry weight in milligram. Numbers of ant contacts (contacts), aggression indices (AI), and Bray–Curtis similarities (BCS) to the average chemical worker profile (centroid) of the ecitophiles’ colony of origin give means and standard deviation (SD). Number in parentheses gives the number of analyzed specimens, which is the same for ant contacts and AI

*Ad* adults, *La* Larvae, *morph.* morphological

We used plastic boxes as observation arenas (50 cm × 30 cm × 25 cm) with a moistened plaster of Paris floor, in which we scratched furrows with a fork to provide hiding spots for ecitophiles. We applied non-perfumed baby powder to the container walls to prevent escape. Immediately after collection, we transferred about 300–400 ant workers, including large soldiers (majors; Fig. 1a-G), medium-sized workers (intermediates; Fig. 1a-H), and small workers (minors; Fig. 1a–I), as well as 50–100 brood items into the observation arena. The collected ecitophiles were kept separately together with some host ants in smaller boxes (15 cm × 10 cm × 5 cm), to which we added leaf litter and moistened crumbled paper as hiding spots. After 6–12 h settling time for laboratory colonies, we started the behavioral assays.

Ecitophiles were introduced one at a time. Each tested specimen was given a settling time of 1 min, after which we noted the behaviors of army ants towards it within a three-minute period. Each physical contact between any body part of the focal test ecitophile and an army ant worker was counted as one contact. Ant behaviors towards ecitophiles included short-lasting contacts such as antennal touch, as well as long-lasting contacts such as grooming. Lasting contacts with the same individual ant were only counted once. The following ant behaviors were observed during contacts between an ant and the focal ecitophile: *no reaction* (ecitophile was not noticed or it was ignored), *grooming*, *antennating*, *seizing*, *carrying* the ecitophile, as well as the ants’ attempt of *chasing*/*snapping* or *stinging* an ecitophile after a short contact. An ecitophile was *seized* if an ant grasped it by the legs or antennae so that it was unable to move away. An ecitophile was *carried* if it was picked up by ants and carried around, or if the ecitophile attached itself to the ant’s body. The behaviors *no reaction* (previously denoted as 'unnoticed' and 'ignored'), *grooming* (or *licking*), *antennating*, and the attempt of *chasing*, *snapping* and *stinging* were defined previously [26, 27, 78]. To measure host aggression against ecitophiles, we calculated an aggression index (AI) by dividing the sum of aggressive interactions (attempt of *chasing*, *snapping*, *stinging,* and *seizing*) by the total number of contacts. We set the AI to zero in cases with no contact between the ants and the focal ecitophile.

In a previous study, we performed a similar behavioral test in a single *E. burchellii* colony focusing on rove beetles of the genera *Ecitophya*, *Ecitomorpha* and *Tetradonia* [38]. That study was conducted in the same army ant-ecitophile community at LSBS in 2014 under similar laboratory conditions [38]. To increase our dataset, we included those data in the analyses reported here. However, data acquisition was slightly different in the rove beetle project, where we only counted the number of total contacts and the number of aggressive behaviors, and we did this for a shorter period of time (one minute instead of three minutes). We thus multiplied the number of contacts and the number of aggressive interactions with three to integrate these data into the present study. As only aggressive behaviors were counted, we could not use this dataset for multivariate analyses of behavioral data (see next paragraph).

We analyzed ant behaviors towards ecitophiles as multivariate data in 'Primer 7' with the software add-on 'PERMANOVA + 1' (Primer-E Ltd., Ivybridge, UK, vers. 7.0.12; [79, 80]). Multivariate behavioral data were analyzed as compositional/proportional data, i.e. each behavioral category was standardized with the total number of contacts for each ecitophile. We used a permutational analysis of variance (PERMANOVA) with 10,000 permutations based on Bray–Curtis similarities [81] to test for differences in ant behavior towards ecitophile species. Ecitophile species, nested within colony, was set as explanatory variable (fixed factor) in the PERMANOVA model design. Colony and observer were set as random factors. Neither colony nor observer showed significant effects (PERMANOVA: pseudo*F* ≥ 2.33, *p* ≥ 0.236). We also used permutational analysis of multivariate dispersions (PERMDISP) to test for the compositional stability of behavioral data [82].

Note that *Vatesus* rove beetles were the only ecitophiles where not only adults but also larvae were present. As army ant behaviors vastly differed towards adult and larval *Vatesus* beetles of the same species (for intraspecific pairwise comparisons between adults and larvae within the host species *E. burchellii*, *E. hamatum* and *E. vagans*, PERMANOVA: pseudo*F* ≥ 33.76, *p* ≤ 0.002; no comparison was possible in other host species), we analyzed the two developmental stages separately in all behavioral tests. For simplicity, we will denote statistical comparisons to be done between species, but readers should be aware that this includes different developmental stages of *Vatesus*.

### Ecitophile body size

To investigate a possible effect of body size on the host’s level of hostile behavior, we measured the dry weight as surrogate of body size in 302 of those ecitophiles used in behavioral assays (Additional file 1). For this, we dried specimens in an oven until weight constancy for at least 48 h at 45 °C and determined their dry weight using a microbalance (Mettler Toledo, XS3DU, USA). Note that the relative size ratio between host ant and myrmecophile might be relevant when investigating its effect on the host’s level of aggression [70]. For three reasons, however, we decided to use the absolute body size as predictor in this study. First, absolute body weight measurements provide definite, non-processed data and they are easier to understand than relative body size relationships. Second, the studied *Eciton* army ants are polymorphic and therefore it is difficult to decide which size class to use as reference size for the ants. Third, size differences between species were small and thus standardizing for host worker size would have not radically changed our analysis (dry weight ranges of *Eciton* workers: *E. burchellii*: 0.417–12.180 mg, N = 85 workers; *E. dulcium*: 0.588–18.182 mg, N = 82 workers; *E. hamatum*: 0.539–12.651 mg, N = 74 workers; *E. lucanoides*: 0.422–8.943 mg, N = 61 workers; *E. mexicanum*: 0.355–6.483 mg, N = 60 workers; *E. vagans*: 0.532–13.375 mg, N = 60 workers). Dry weight measurements are given in Additional file 1 and are summarized in Figure S1–f of Additional file 2.

### CHC profiles of army ants and ecitophiles

We analyzed the cuticular hydrocarbon (CHC) profiles of host army ants and ecitophiles to investigate a possible host resemblance in chemical recognition cues. We extracted CHCs of a minimum of 10 majors, 10 intermediates, 10 minors, and 10 ecitophile specimens per species (if available) from a total of 14 army ant colonies (including those used in behavioral tests): two *E. burchellii* colonies (plus one published previously [38]), three *E. dulcium* colonies, three *E. hamatum* colonies, two *E. lucanoides* colonies, two *E. mexicanum* colonies, and two *E. vagans* colonies (Table 1). The final sample size of 30 worker CHC profiles per colony was not reached in all cases since some extraction vials broke during the transport from Costa Rica to Germany (Table 1).

Those ecitophiles tested in behavioral assays were extracted within a period of 1–15 min after the test, while ant workers were extracted at the end of behavioral tests. Specimens were transferred individually into 1.5 ml vials with PTFE-coated caps (Agilent) and submerged for 10 min at room temperature in 200 μl n-hexane (98% purity for gas-chromatography; Sigma-Aldrich). Subsequently, we removed specimens from hexane and preserved them in absolute ethanol for genetic and morphological analyses [75]. Hexane was then evaporated at room temperature in a fume-hood at LSBS, before transporting CHC extracts to Germany.

In the laboratory at the TU Darmstadt, Germany, we re-dissolved the CHC extracts in 40 µl n-hexane containing octadecane as internal standard (10 ng octadecane per 1 µl hexane). We then transferred 20 µl of the samples into conical glass inlets and analyzed them with a QP 2010ultra GC–MS (Shimadzu, Japan). The gas chromatograph was equipped with a ZB-5MS fused silica capillary column (30 m × 0.25 mm ID, *df* = 0.25 μm) from Phenomenex (USA). We used an AOC-20i autosampler-system from Shimadzu to inject 1 μl sample aliquot into a programmed temperature vaporizing split/splitless-injector in splitless mode (Optic MultiMode Inlet 4, GL Sciences, Netherlands). The initial injection temperature was 50 °C (5 s hold), which was increased to 300 °C with a heating-rate of 50 °C/s and a subsequent hold for 59 min. We used hydrogen as carrier-gas with a flow rate of 1.3 ml/min. The GC oven temperature was raised from an initial 60 °C for 1 min, to 320 °C with a heating-rate of 5.5 °C/min followed by an isothermal hold at 320 °C for 10 min. Electron ionization mass spectra were recorded at 70 eV with a scan rate of 2 scans per second from *m/z* 40 to 650. We kept the ion source of the mass spectrometer and the transfer line at 230 °C and 300 °C, respectively.

We identified chemical compounds by examining the CHCs’ *m/z* fragmentation patterns and gas chromatographic retention indices (RI) using extracts of 10–20 pooled ant workers for each *Eciton* species. RIs were calculated with an alkane standard mixture (C7-C40 dissolved in hexane; Sigma-Aldrich, Germany) using the method of van den Dool and Kratz [83]. We assigned the structural identities of methyl-branched alkanes using diagnostic ions and RIs [84, 85], and we applied iodine-catalyzed dimethyl-disulfide derivatization to determine double bond positions in alkenes and alkadienes [86]. We did not determine the configurations of double bonds.

For compositional data analyses we excluded those GC–MS runs in which we did not detect a single compound (26 phorid flies, 10 *Vatesus* larvae, seven *Myrmedonota* specimens, and three ptiliid beetles; Additional file 1). Still, our compositional dataset contained many zeros, which might have been partly caused by CHC detection limitations in tiny arthropods (e.g., phorid flies and ptiliid beetles). To account for such a potential bias, we set CHCs below a threshold of 1% of the total CHC amount per GCMS run to a value of zero. Furthermore, we used the R function ˈmultLRˈ (Multiplicative Lognormal Replacement) implemented in the R package ˈzCompositionsˈ [87] to replace zeros based on a multiplicative log-normal imputation of left-censored data, thus preserving the samples’ multivariate compositional properties.

The resulting CHC compositional/proportional data were analyzed in Primer 7 and visualized using NMDS ordination plots. To test for compositional differences and compositional stability of CHC profiles between different *Eciton* species and between different ecitophile species, we used a PERMANOVA and a PERMDISP, respectively. The PERMANOVA design to test for CHC profile differences between army ant species was as follows: army ant species was used as fixed factor, colony nested in species as random factor, and worker type (minor, intermediate, and major worker) nested in colony as random factor. A second PERMANOVA design was used to test for differences between CHC profiles of ecitophile species: ecitophile species (fixed factor) nested in colony (random factor). Like in behavioral assays, we treated *Vatesus* adults and *Vatesus* larvae as distinct categories. In addition, we used the Bray–Curtis (BC) similarity [81] to the average colony worker profile (i.e., the colony centroid) to describe chemical similarities of ecitophiles to their host colony (see also [26, 27]). BC similarity values can scale between 0 in specimens with no overlap in CHC profile to host workers to 100 in specimens with a perfect match of the chemical profile.

Furthermore, we investigated a possible influence of CHC concentrations on host hostility and the transfer of a labeled CHC from the cuticle of ant workers to myrmecophiles. These analyses are presented and discussed in Additional file 3.

### Comparisons across ecitophile phenotypic traits

We included the categorial variable 'morphological gestalt' in our models to explore whether an ecitophile’s gestalt relates to its level of received ant aggression, as already predicted by Wasmann in 1895 [43]. In this study, we distinguished five types of morphological gestalt: protective, myrmecoid, staphylinid-like, phorid-like, and larval-shaped (Fig. 1a–A–F). Note that the morphological gestalt went hand in hand with a rather close phylogenetic relationship of ecitophiles in all but the specimens of the protective gestalt (Table 2). This means we did not have phylogenetically independent replicates for most gestalts. The reader should thus be aware that we cannot disentangle the influence of morphological gestalt from possible confounding factors caused by close phylogenetic relationship. Table 2 gives an overview of phylogenetic relationships and the morphological gestalt of each species, allowing the reader to assess the phylogenetic diversity for each morphological gestalt.

We combined data on body size, morphological gestalt, and accuracy in CHC host mimicry to explore traits that potentially govern the degree of ant aggression against ecitophiles. We fitted generalized linear mixed-effects models in R using ant aggression towards ecitophiles as binomial response variable (cbind(); number of aggressive interactions vs. number of non-aggressive interactions) and the ecitophiles’ dry weight (square-root-transformed), morphological gestalt, and accuracy in host mimicry (BC similarity to average worker profile) as fixed variables. *Eciton* colony ID, ecitophile species, and observer were fitted as random-effects terms. We used a type-III analysis-of-variance using the chi-square test (Anova() as implemented in the package 'lme4') [88]. We first tested all possible interaction terms of the three fixed variables and deleted non-significant ones from the model [89]. The resulting model had lower AIC/BIC values than the basic model without interaction terms. The inspection of the model’s residual distribution detected no significant problem, which we examined using the function plotResiduals() as implemented in the package 'DHARMa' [90].

The 'protective gestalt' (also denoted as “Trutztypus” or “Schutzgestalt” by Wasmann and others; [2, 43–45]) includes teardrop-shaped species and species with a globular-protective morphology (Fig. 1a-B,C). The present study included six beetle species and one silverfish species of the teardrop-shaped gestalt (also denoted as “Schutzdach-Typus” or “*Limulus*-Gestalt” by Wasmann [43]) (Table 2; Additional file 1). These limuloids are characterized by laterally expanded body plates (pronotum and elytra in beetles; pro-, meso- and metanotum in silverfish) that form a protective shield, underneath which the head and most appendages can be retracted [2, 51]. Like teardrop-shaped specimens, histerid beetles and the single water scavenger beetle have a protective morphology by possessing a heavily sclerotized, robust, and broadly globular body [14, 45, 56]. In histerids, the appendages and the head are retractable into anatomical cavities, leaving the ants little to no point of attack (denoted as “Trutztypus” [45]; for more details see [14, 45, 53, 56]). Our study included seven histerid species and one water scavenger beetle (Table 2; Additional file 1). The 'myrmecoid gestalt' (denoted as “Mimikry Typus” by Wasmann [43]) possesses no obvious protective structures but instead anatomically resembles ant workers by having a narrowed waist with expanded abdomen (petiolate abdomen), geniculate antennae, and long legs relative to body size (e.g., [14, 42, 47]; Fig. 1a-A). Five species of myrmecoid beetles were included in the present study (Table 2; Additional file 1). Species possessing the typical 'staphylinid-like gestalt' (denoted as “indifferent Typus” by Wasmann [43]) have a slender, elongate body shape, short elytra, and a flexible abdomen without expressing petiolate abdominal structures like in myrmecoids (see also [14, 91]; Fig. 1a-F). They have the general appearance of staphylinid beetles and differ little in their morphology from their free-living relatives. This study included eight species with such a gestalt (Table 2). We defined the 'larval-shaped gestalt' to have a slender, elongate body shape with a weak level of sclerotization and pronounced body setation (see [76]). This gestalt solely included the larvae of three *Vatesus* beetle species (Fig. 1a-D; Table 2; Additional file 1). Lastly, all phorid fly species were defined here as the 'phorid-like gestalt' (Fig. 1a-E; Table 2), which was characterized by extremely elongated legs relative to the rest of the body, as well as a somewhat globular appearance with a weak level of sclerotization compared to beetles (for details see [2, 92]). Six species of phorid flies were included in this study (Table 2; Additional file 1).

## Results

### Army ant behavior towards ecitophiles

We found a diverse spectrum of strategies in ant guests to cope with the potentially aggressive army ant hosts. While some species had frequent contact with host workers, others had only few to no contacts (range = 0–208 contacts per 3 min, N = 314 specimens; Fig. 1b). In most host-ecitophile encounters, the ants showed *no reaction* (11,091 out of a total of 12,783 contacts, N = 253 specimens; Additional file 2: Fig. S1a; e.g., Additional file 4). Yet, when considering all behaviors as compositional data, army ants reacted differently towards different ecitophile species (PERMANOVA: pseudo*F* = 3.651, *p* = 0.005).

Ants attacked ecitophiles in 5% of encounters (988 aggressive contacts out of a total of 18,036 contacts, N = 314 specimens, Additional file 1). Most ecitophiles were not or only rarely attacked by host ants (Fig. 1c), yet the following species were attacked on a regular basis: rove beetles of the genera *Vatesus* (adults; mean ± SD: 14 ± 12 aggressive behaviors per 3 min, N = 43 specimens; Fig. 1c; Additional file 5), *Tetradonia* (mean ± SD: 3 ± 5 aggressive behaviors per 3 min, N = 50 specimens; Fig. 1c), and *Proxenobius* (mean ± SD: 7 ± 5 aggressive behaviors per 3 min, N = 5 specimens; Fig. 1c); the ptiliid beetle *Cephaloplectus mus* (mean ± SD: 3 ± 3 aggressive behaviors per 3 min, N = 16 specimens; Fig. 1c), the histerids of the genus *Euxenister* (mean ± SD: 5 ± 9 aggressive behaviors per 3 min, N = 5 specimens; Fig. 1c),  and the silverfish *Trichatelura manni* (mean ± SD: 4 ± 8 aggressive behaviors per 3 min, N = 32 specimens; Fig. 1c). Notably, no ecitophile was killed by the ants in behavioral tests.

In the following, we briefly highlight some notable host-ecitophile interactions. Species of the genera *Ecitophya*, *Ecitomorpha*, *Euxenister* and *Nymphister* were regularly groomed/licked by host ants, indicating a high level of integration into the army ant society (mean *grooming* ± SD per 3 min: *Ecitophya* = 11 ± 1, N = 2; *Ecitomorpha* = 5 ± 3, N = 3; *Euxenister* = 9 ± 13, N = 5; *Nymphister* = 4 ± 2, N = 11; Additional file 2: Fig. S1b; Additional files 6–8). *Carrying* was rare and only observed in the ptiliid beetle genus *Cephaloplectus* (N = 1 specimen), as well as in the histerid genera *Cheilister* (N = 1 specimen), *Euxenister* (N = 2 specimens), *Nymphister* (N = 1 specimen), and *Psalidister* (N = 1 specimen). Similarly, *seizing* was uncommon and occurred only in *Euxenister* histerid beetles (N = 3 specimens; Additional file 9), and rove beetles of the genera *Tetradonia* (N = 3 specimens), *Vatesus* (N = 1 adult specimen), and *Proxenobius* (N = 1 specimen). Table S1 of Additional file 2 provides an overview of ant behaviors towards different ecitophile species and supplemental videos document a few representative host-symbiont interactions (Additional files 4–10).

### CHC profiles of *Eciton* army ants

Army ants possessed relatively simple cuticular hydrocarbon profiles, which were dominated by the following compound classes: alkanes, alkenes, alkadienes, and mono-methylated alkanes. In total, we detected 89 chemical compounds in *Eciton* army ants, of which 30 CHCs remained for data analysis after exclusion of low abundance peaks (see Additional file 1 for a compound list).

Army ant species had distinct CHC profiles (PERMANOVA: pseudo*F* = 217.98, *p* < 0.001) and formed clusters in NMDS ordination plots (Additional file 2: Fig. S2a). Some species showed more variable CHC profiles than others (PERMDISP: F_5,404_ = 24.73, *p* < 0.001; Additional file 2: Fig. S2a). Notably, CHC profiles of the three sister species *E. burchellii*, *E. hamatum*, and *E. lucanoides* clustered closely together in a NMDS ordination plot (Additional file 2: Fig. S2a) as they shared the same major compounds: C21, C23-9-ene, and C23 (Additional file 1). Yet, the three species differed significantly in CHC profile composition (PERMANOVA of all pairwise comparisons: pseudo*F* ≥ 5.91, *p* < 0.001; Additional file 2: Fig. S2b,c).

We did not detect clear compositional differences between colonies of the same species (PERMANOVA: N_1_ = 16 colonies, N_2_ = 410 workers, pseudo*F* = 1.60, *p* = 0.083), but the statistical power to detect such differences was low due to a limited number of analyzed colonies per species (Table 1). Intracolonial worker types, i.e. minor, intermediate and major workers, differed in CHC composition (PERMANOVA: N_1_ = 16 colonies, N_2_ = 120 minors, 148 intermediates, 142 majors; pseudo*F* = 13.95, *p* < 0.001; see also [38]).

### CHC mimicry accuracy of ecitophiles

**Fig. 2:**
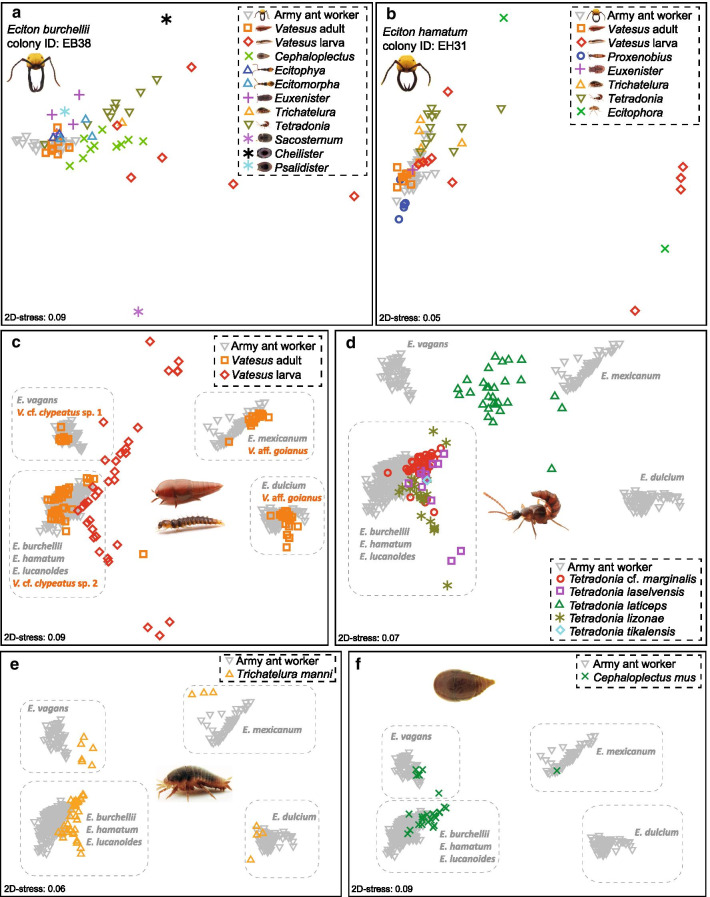
CHC resemblance of ecitophiles to host workers. NMDS plots visualizing CHC profile similarities between host ants and ecitophiles in one colony of **a** *Eciton burchellii* and one colony of **b** *E. hamatum* (for other colonies see Additional file 2: Fig. S3). Chemical profiles of all *Eciton* workers in the community and **c** *Vatesus* adults and larvae, **d** *Tetradonia* beetles, **e** the silverfish *Trichatelura manni*, **f** and the ptiliid beetle *Cephaloplectus mus*. Grey dashed boxes contain host ants and those ecitophiles that were collected in colonies of that host(s), except for *Vatesus* larvae in **c** and *Tetradonia laticeps* rove beetles in **d**, which were not assigned to host ants via grey dashed boxes. *Vatesus* cf. *clypeatus* sp. 1 larvae were collected from *E. vagans* (N = 14 specimens), *Vatesus* cf. *clypeatus* sp. 2 larvae from *E. burchellii* (N = 13 specimens) and *E. hamatum* (N = 7 specimens), and *Vatesus* aff. *goianus* larvae from *E. mexicanum* (N = 5 specimens). *Tetradonia laticeps* rove beetles in **d** were collected from *E. mexicanum* (N = 12 specimens) and *E. dulcium* (N = 17 specimens) colonies

We exclusively detected CHCs in ecitophiles that were also detected in host workers. The degree of host similarity in CHC profiles varied substantially between ecitophile species, with some species mimicking the hosts’ CHC profiles with high accuracy (Figs. 1d, 2). Consequently, CHC profiles differed between ecitophile species from the same host colony (PERMANOVA: pseudo*F* = 8.02, *p* < 0.001), with some ecitophile species showing more variable CHC profiles than others (PERMDISP: F_43,459_ = 13.05, *p* < 0.001; Fig. 2 and Additional file 2: Fig. S3).

Similarities of CHC profiles between ecitophiles and host ants are summarized in Fig. 1d. Highest CHC similarities to colony profiles were detected in species of the rove beetle genera *Ecitomorpha* (median BC similarity = 91, N = 27), *Ecitophya* (median BC similarity = 94, N = 25), *Proxenobius* (median BC similarity = 92, N = 13), *Vatesus* adults (median BC similarity = 93, N = 79), and in species of the histerid genus *Nymphister* (median BC similarity = 90, N = 26). Weak CHC host resemblance was detected in all phorid fly genera (median BC similarity: *Ecituncula* = 55, N = 14; *Ecitophora* = 35, N = 15; *Thalloptera* = 34, N = 6), *Vatesus* larvae (median BC similarity = 36, N = 42), and *Myrmedonota* rove beetles (median BC similarity = 34, N = 18).

Notably, CHC profiles showed considerable intraspecific differences in several multi-host species: adults of the rove beetles *Vatesus* aff. *goianus* (Fig. 2c) and *V.* cf. *clypeatus* sp. 2 (Additional file 2: Fig. S4), as well as the silverfish *Trichatelura manni* (Fig. 2e) and the ptiliid beetle *Cephaloplectus mus* (Fig. 2f). Specimens of those species chemically resembled those host species from which they were collected from, providing circumstantial evidence for CHC acquisition from host ants (see also Additional file 3: experiment 2).

### Comparisons across ecitophile phenotypic traits

**Fig. 3:**
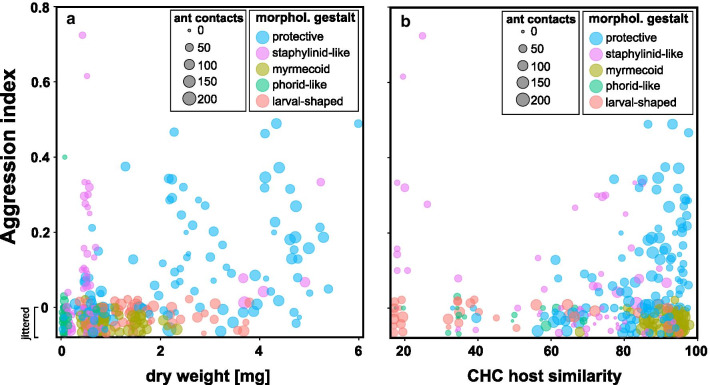
Ant aggression towards ecitophiles in relation to their **a** dry weight and **b** CHC host similarity. The aggression index gives the sum of aggressive interactions (attempt of *chasing*, *snapping*, *stinging,* and *seizing*) divided by the total number of contacts. CHC host similarity is given as Bray–Curtis similarity of a given ecitophile to the average worker profile of its colony of origin. Colors depict the morphological gestalt of ecitophiles and data point size indicates the number of contacts

The ecitophiles’ dry weight had a significant effect on the level of received host aggression (Table 3), in that ant aggression increased with increasing dry weight (Fig. 3a). Small ecitophiles such as phorid flies (genera *Ecitophora*, *Ecituncula*, *Thalloptera*), ptiliid beetles of the genus *Limulodes* and staphylinid beetles of the genus *Myrmedonota* were rarely attacked by ants (Table 2). For instance, only five specimens were attacked out of those 55 specimens having a dry weight of less than 0.30 mg (phorid flies: 39 specimens; ptiliid beetles: 5 specimens; rove beetles: 11 specimens; Additional file 1). In comparison, large ecitophiles with a dry weight above 3.00 mg (histerid beetles: 3 specimens; silverfish: 8 specimens; rove beetles: 40 specimens) were more often aggressed: 40 specimens out of 51 were attacked at least once (Additional file 1).

**Table 3:** Predictors of ant aggression towards ecitophiles

Predictor variables	Chi sq	*Df*	P
Dry weight (sqrt)	41.146	1	0.001
Morphological gestalt	34.497	4	0.001
CHC host similarity	00.003	1	0.958
Morphological gestalt * CHC host similarity	21.345	4	0.001

Results of the general linear mixed effects model with aggressive contacts vs. non-aggressive contacts as binomial response variable

*Df.* degrees of freedom, *Chi sq.* Chi square, *sqrt* square root transformed data

The morphological gestalt was also a significant predictor of host hostility against ecitophiles (Table 3). Ant aggression was primarily directed towards ecitophiles with a protective and a staphylinid-like gestalt, while ecitophiles of the phorid-like, larval-shaped, and myrmecoid gestalt were rarely attacked (Fig. 3 and Additional file 2: Fig. S5).

Unexpectedly, we found no clear overall effect of CHC host similarity on host aggression (Table 3), but a significant interaction between CHC host similarity and morphological gestalt. This pattern was mainly driven by an increase of host aggression with increasing CHC host similarity in ecitophiles of the protective gestalt (Fig. 3B and Additional file 2: Fig. S5). When considering other types of morphological gestalt, an increasing CHC host similarity rather led to decreasing ant aggression in staphylinid-like specimens, while specimens of the myrmecoid, phorid-like and larval-shaped gestalt were rarely attacked, irrespective of their CHC host similarity (Fig. 3B and Additional file 2: Fig. S5).

## Discussion

Here we documented host behavior towards symbionts in a community of army ants and their entourage of arthropod guests, and evaluated three guest traits as possible aggression triggers. In contrast to our expectations, we did not detect a clear relationship between chemical mimicry accuracy and the frequency of host attacks. We thus conclude that there must be other aspects of the ecitophiles’ phenotypes that explain why ants treat them differently. A wide range of exploitation strategies exist among social insect symbionts, and morphological, behavioral, chemical and vibroacoustical adaptations have been documented [23, 27, 37–41]. We here focused on two additional morphological aspects of a guest’s phenotype—the body size and the morphological gestalt. In the following, we discuss a possible role of these traits in eliciting hostile ant behavior, considering that phenotypic guest traits might be correlated with each other (e.g., body size and morphological gestalt in phorid flies), and with phenotypic traits not explicitly examined in the present work (e.g., flight response of limuloid guests).

First, it is important to realize that attacks were overall rare, likely because the ecitophiles we collected during host emigrations had already infiltrated the colonies of army ants [75]. The guests had the opportunity to seek physical contact with the ants prior to our behavioral tests, allowing them to acquire the colony-specific gestalt odor from the workers ([26, 30, 34, 93, 94]; see also Additional file 3: experiment 2). Indeed, many guests had physical contact with workers in laboratory nests (Additional files 4–10) and possessed high levels of chemical mimicry accuracy. This in turn might partly explain the overall low level of host aggression (e.g., [38] and references therein). The weak defensive host response observed in this study is consistent with the popular metaphor describing social insect symbionts as living in a 'safe haven inside an enemy-free fortress' (e.g., [39, 95–97]). As generally predicted for social insect symbionts [1, 2, 4, 16], living inside or close to the bivouacs of army ants might protect ecitophiles from their own predators, parasites, and parasitoids. Nonetheless, we consider life inside or close to the bivouacs of army ants to be risky for arthropod guests. While no individual was killed in this study, occasional killings do occur in ecitophiles [19, 43, 49, 55, 98] and are also common in other ant-myrmecophile systems (e.g., [26, 61, 70, 99]). The fortress guards themselves—the ant workers—certainly represent an often-underestimated risk to the life of social insect guests.

The army ant workers primarily attacked ecitophiles with either a protective or a staphylinid-like gestalt, except for diminutive species of these gestalts (Table 2). Guests of other gestalts received minimal levels of aggression, which indicated that the morphological gestalt is relevant in context of host defensive responses, as indicated but not explicitly tested previously [2, 4, 13, 14, 19, 55, 68, 78]. However, this interpretation needs to be treated with caution because our study lacked phylogenetically independent replicates in all morphological gestalts except for the protective gestalt (Table 2).

Ecitophiles with a protective gestalt had vastly different phylogenetic backgrounds (Table 2). Evidently, morphological shielding protected the limuloid-shaped guests during ant attacks (see also [13, 14, 26, 49, 98]; Additional file 5). Limuloids frequently showed swift escape movements during ant encounters (e.g., Additional file 5), which might be primarily responsible for the elevated aggression towards these guests—instead of the limuloid gestalt per se. We frequently observed this flight behavior in the silverfish *Trichatelura manni*, the three *Vatesus* rove beetles, and the ptiliid beetle *Cephaloplectus mus,* as well as in previous studies of a limuloid silverfish guest of *Leptogenys* army ants [26, 27, 93]. Presumably, swift movements elicit ant aggression because they are typical for intruders or prey (see also [27, 100–102]).

In contrast, the tortoise-like histerid beetles and the water scavenger beetle, both also possessing a protective gestalt, moved slowly compared to limuloids and the host ants. Their primary countermeasure against host aggression was their robust, heavily sclerotized anatomy, which protected them from injuries during attacks (see also [14, 45, 53, 59, 60]; Additional file 9). In addition, some Neotropical histerids possess exocrine glands at prominent patches of setae (i.e., trichomes). Secretions from these glands can alter host behavior [14, 45, 56]. Ants have been observed to intensively lick these trichomes, facilitating peaceful host-myrmecophile interactions [4, 14, 16, 68, 103]. In the present work, species of the genera *Aphanister*, *Nymphister* and *Euxenister* possessed prominent setation [56], and these species were also intensively *groomed*/*licked* by ant workers in laboratory nests (Additional file 2: Fig. S1; Additional file 8). For instance, *Euxenister caroli* possesses several trichome patches on the abdomen and along the carinae of the pronotum and elytra, as well as setation of unknown function on various other body parts [60, 104]. Based on histological sections, Reichensperger reported that these beetles have exocrine glands in the legs, the carinae of the pronotum and elytra, the forehead, and the pygidium [60, 104, 105]. While trichome-associated exocrine glands have long been known [37, 45], their chemical exudates have not been intensively studied yet [16], clearly offering plentiful opportunities for future research.

Besides ecitophiles of the protective gestalt, rove beetles with a typical staphylinid-like body shape were regularly attacked by army ants. They were represented by seven species of three genera: *Tetradonia*, *Myrmedonota* and *Proxenobius*. The five *Tetradonia* species are close relatives and might thus share many other aspects of their phenotypes besides their staphylinid-like gestalt. The genus *Myrmedonota* belongs to the same staphylinid tribe as *Tetradonia*, 'false Lomechusini'—a group of Neotropical aleocharine rove beetles formally placed in the tribe Lomechusini [106]. Species of these two genera were relatively small (dry weight ≤ 0.82 mg; N = 130; Table 2), swiftly moving, and non-integrated aleocharine rove beetles with efficient escape responses during attacks—probably the most common strategy of staphylinids to counteract ants [2, 14, 43]. As discussed above for limuloid guests, these swift escape responses might be an important factor eliciting host aggression irrespective of the guest’s morphological gestalt. Additionally, these beetles evaded ant encounters by hiding in small cavities of the observation arena, a typical habit of many, if not most non-integrated myrmecophilous staphylinids [2, 38, 68, 78, 107, 108]. In contrast, *Proxenobius borgmeieri* belongs to the rove beetle subfamily Staphylininae, and it is, as many members of this subfamily [14], relatively large (mean dry weight = 4.46 mg, N = 13). Hiding in the observation arena was thus hardly possible. This ecitophile had frequent contact with host ants and it was frequently attacked. Swift movements and possibly the use of defensive chemicals, as indicated by a bent abdomen directed at ant workers during aggressive encounters (see also [74]), supposedly saved this ecitophile from being captured and killed. Generalized myrmecophilous staphylinids such as *Proxenobius* [42] and species of the genera *Tetradonia* [109] and *Myrmedonota* [110] usually possess exocrine abdominal glands (e.g., the 'tergal gland' [14]), which contain defensive irritants to repulse or baffle ants during aggressive encounters [14, 19, 41, 111–113].

While ecitophiles with a protective and a staphylinid-like gestalt were commonly attacked, those with a myrmecoid, a larval-shaped, and a phorid-like gestalt were barely attacked. Of these, myrmecoids were the only behaviorally fully integrated ecitophiles (sensu [2]), meaning they were incorporated into the host society by their own and their hosts’ behavior. They usually sought contact with host workers with whom they peacefully interacted (Additional file 6). Ants frequently groom nestmates [4, 24, 114], and myrmecoids were likewise groomed by army ant workers (see also [19, 38]). This high level of social integration might be attributed to the multiple adaptations to myrmecophily in these beetles, among them the ant-mimicking gestalt, a high accuracy in chemical host mimicry, behavioral adaptations and, in certain species, special exocrine abdominal glands that are suspected to facilitate peaceful social interactions [14, 38, 47, 48, 50]. Based on circumstantial evidence, several authors hypothesized that mimicking the ants’ body shape and cuticular sculpture might exploit a tactile recognition system [2, 27, 38, 45, 46, 115, 116]. This idea was first formulated by Erich Wasmann as 'tactile mimicry', and later coined 'Wasmannian mimicry' by Carl Rettenmeyer [44, 46]. Wasmann speculated that, besides resembling the host in chemical odor, ant guests also need to exhibit anatomical similarities to host ants for achieving high levels of social integration. In other words, successful anatomical mimicry implies that the body shape and the surface structure between a Wasmannian mimic and a nestmate worker is indistinguishable to an ant’s tactile inspection. This hypothesis has largely been put aside in studies of social insect symbionts, as most studies have focused on behavioral, vibroacoustical and chemical integration mechanisms [26, 30, 78, 117]. However, a recent study has drawn renewed attention to this subject because it demonstrated that a group of socially parasitic ants closely resemble their host ants in size and shape, suggesting that morphological cues, besides olfactory ones, might be surveilled by ants in order to distinguish nestmates from foes [118, 119]. This provided evidence in favor of Wasmann’s hypothesis, suggesting that the myrmecoid gestalt might be an important mechanism in the exploitation of army ant societies. Noteworthy, the myrmecoid gestalt has evolved at least 12 times independently within ant-associated aleocharine rove beetles, suggesting that it is indeed of high adaptive value [47].

Our community-based study on *Eciton* guests also included beetle larvae. Larvae infiltrating the nests of ants  exist in diverse insect taxa, among others in beetles, butterflies, and flies, and various adaptations to myrmecophily have been documented for these immature ant guests [4, 13, 29, 120–125]. In the present study, this morphological gestalt solely included three species of the tachyporine rove beetle genus *Vatesus*. Accordingly, we cannot conclude that the larval-shaped gestalt itself was responsible for low aggression levels, but that specific phenotypic traits of *Vatesus* larvae inhibited ant aggression. Like myrmecoids, *Vatesus* larvae had frequent contacts with host ants (Fig. 1c) but, instead of being groomed, they were mostly not recognized and if so, they were briefly antennated by the ants (Additional file 2: Fig. S1) and then left alone. *Vatesus* larvae were not socially integrated guests, but still appeared to be mostly tolerated by the ants. This is remarkable as most specimens were relatively large (mean dry weight = 1.80 mg; range = 0.37–3.79 mg; N = 49) and their resemblance of chemical host recognition cues was comparably weak (Fig. 1d; Table 2). As commonly suggested for social insect symbionts in general [25, 94, 94, 126, 127], the minimal concentrations of CHCs in *Vatesus* larvae might have hampered the host’s chemical recognition (chemical hiding sensu [32]; chemical insignificant sensu [24, 36]; Additional file 3: experiment 1). Further, Rettenmeyer speculated that the numerous and long macrosetae protruding from all sides of the body (Fig. 1a-D) protect *Vatesus* larvae from host attacks [49]. This seems plausible as setae and spines are known to be used as mechanical anti-predation mechanisms by various arthropods, including larvae of moths and beetles [128–130].

Contrary to myrmecoids and *Vatesus* larvae, host contacts were overall rare in specimens of the phorid-like gestalt (Fig. 1b). This gestalt included solely flies from the family Phoridae (Table 2), so that its members shared many additional phenotypic traits besides their morphology. For instance, these miniature ecitophiles efficiently avoided contacts with ants by moving away promptly in their typical stop-and-go, zigzag manner, often before physical contact even occurred (see also [49, 131]). During contacts, they were mostly not recognized by the ants, which we attribute primarily to their diminutive size and their fast and scuttling gait [132], rather than to their morphological gestalt per se. The minimal level of aggression towards diminutive phorids thus agrees with our general finding that smaller-sized ecitophiles were less often attacked. In fact, especially tiny ecitophiles elicited little to no host aggression, agreeing with previous suppositions that diminutive myrmecophiles such as mites, phorids, ptiliids, and springtails usually stay undetected in ant nests [4, 16, 25, 43, 68, 73]. Apparently, with a diminutive body size, myrmecophiles enter an enemy-free space [133] that not only protects the intruders from own predators, parasites and parasitoids [4, 18, 134], but also from the attacks of host ants in otherwise well defended colonies.

To persist in social insect colonies, the use of chemical mimicry (sensu [32]) as integration strategy is often assumed to be of major importance for social insect guests [30, 34]. We found that many ecitophiles indeed mimicked their host recognition cues, and we expected to find a higher level of host aggression towards those ecitophiles with lower accuracy in CHC mimicry (e.g., [26]). While this relationship appeared to exist in rove beetles of the staphylinid-like gestalt, this was not the case in ecitophiles of other types of morphological gestalt—with myrmecoids, phorids and *Vatesus* larvae receiving little to no aggression, irrespective of their accuracy in mimicking host CHC profiles. We assume that additional adaptations, such as the ones discussed above, might free these ecitophiles from the need of accurately resembling the host colony odor to avoid host attacks. This does not exclude the possibility that a higher accuracy in chemical mimicry might still provide some benefits to these ecitophiles, such as a reduced frequency of inspection by host ants (e.g., [27]).

Similar to our findings, other studies on social insect guests failed to find a clear relationship between CHC host similarity and host aggression [27, 135]. For instance, the spider *Sicariomorpha maschwitzi* (formerly *Gamasomorpha maschwitzi*; [136]), a guest of *Leptogenys* army ants, was not attacked by host workers and remained socially well integrated despite experimentally reduced CHC host similarity [27]. While the spider did not depend on accurate chemical host mimicry to avoid host aggression, a silverfish infesting the same host species did so [26, 27, 61]. Apparently, the two myrmecophiles relied differently on accurate chemical mimicry to persist within colonies of the same host ant species [27, 61]. Another example is given by the diverse guest fauna of mound-building red wood ants, where most guests did not mimic the chemical recognition cues of their host [135]. Here, the haystack-like structure and large size of the dome-shaped nest mounds were assumed to provide plentiful hiding spots, presumably freeing those myrmecophiles from the need of mimicking chemical host recognition cues [135]. The degree of dependency on chemical mimicry thus seems to vary between species [27], depending on the biology of the hosts (e.g., haystack-like nest dome) and on that of the guests (e.g., morphological and behavioral adaptations, chemical weaponry/trickery, acoustic mimicry).

Unexpectedly, specimens mimicking the chemical host recognition cues with higher accuracy were more often attacked in ecitophiles of the protective gestalt (Additional file 2: Fig. S5)—a pattern that has not yet been detected in other social insect symbionts as far as we know. We have no evidence-based explanation for this (Additional file 3: experiment 1), and it remains for future studies to verify whether this is a more common pattern in social insect symbionts with a protective gestalt, for example by monitoring the ants’ behaviors towards symbionts with experimentally reduced CHC host similarities [26, 27].

To summarize, the present study highlights the plurality of interactions between symbionts and their social insect hosts in an army ant-guest community. We provided correlative evidence that multiple phenotypic traits of myrmecophiles are involved in social insect exploitation. We failed to find a clear relationship between CHC host similarity and host hostility, and thus concluded that there must be other phenotypic traits explaining the differing aggression towards symbionts. Body size was a good predictor of host aggression in phylogenetically unrelated species, in that diminutive forms received little to no ant aggression. While we also presented evidence that the symbionts’ morphological gestalt might be a valuable predictor of ant aggression, symbiont phylogeny was certainly a confounding factor, and phylogenetically independent replicates were missing in all but the protective gestalt.

## Conclusion

Research on social insect guests has often focused on a single or a few guest traits when studying host infiltration/integration mechanisms, predominantly chemical integration strategies and/or the hosts’ and the symbionts’ behaviors (e.g., [28, 31, 38, 78, 94, 97, 137, 138]). We expanded the inventory of commonly studied guest traits by including two easily recordable traits: the body size and the morphological gestalt. Integrating these traits suggested that focusing on a single or a few traits likely falls short in explaining host exploitation mechanisms, supporting previous findings in other social insect-symbiont systems (e.g., [21, 23, 139, 140]). We thus conclude that future work should best integrate multiple phenotypic guest traits synergistically in a comparative framework to point towards possible proximate mechanisms underlying social insect exploitation, which can then be studied in more detail with controlled manipulative experiments.

## Supplementary Information

**Additional file 1**. Raw data of dry weight measurements and behavioral assays as well as CHC compositional data of *Eciton* workers.

**Additional file 2**. The file contains supplemental figures and tables.

**Additional file 3**. Supplemental experiments addressing the influence of CHC concentration on host aggression and the acquisition of CHCs by ecitophiles.

**Additional file 4**. Video of ptiliid beetle *Cephaloplectus mus* walking underneath an *Eciton burchellii* worker, not evoking a behavioral response of the worker (ant behavior *no reaction*).

**Additional file 5.** Video of limuloid rove beetle *Vatesus* cf. *clypeatus* sp. 1 being aggressed (*chased/snapped*) by *Eciton dulcium* host workers.

**Additional file 6**. Video of myrmecoid rove beetle *Ecitophya simulans* being licked by an *Eciton burchellii* host worker and simultaneously licking a host worker itself.

**Additional file 7**. Video of histerid beetle *Nymphister kronaueri* attached to an *Eciton mexicanum* worker in its typical phoretic pose. The beetle was groomed/licked by another host worker.

**Additional file 8**. Video of *Euxenister caroli* histerid beetle being groomed/licked by *Eciton burchellii* host worker.

**Additional file 9**. Video of *Eciton hamatum* worker grabbing and holding a leg of the histerid beetle *Euxenister wheeleri* (ant behavior *seizing*).

**Additional file 10**. Video of *Euxenister wheeleri* histerid beetle sitting on an injured *Eciton hamatum* major. The beetle intensively rubbed its legs on the ant’s and its own body, presumably to transfer cuticular hydrocarbons.

## Data Availability

The datasets supporting the conclusions of this article are included within the article and its additional files.
