# Correction to: Multiple phenotypic traits as triggers of host attacks towards ant symbionts: body size, morphological gestalt, and chemical mimicry accuracy

**DOI:** 10.1186/s12983-021-00443-8

**Published:** 2022-01-11

**Authors:** Christoph von Beeren, Adrian Brückner, Philipp O. Hoenle, Bryan Ospina-Jara, Daniel J. C. Kronauer, Nico Blüthgen

**Affiliations:** 1 Technical University of Darmstadt, Darmstadt, Germany; 2 Division of Biology and Biological Engineering, California Institute of Technology, Pasadena, USA; 3 Department of Biology, University of Valle, Cali, Colombia; 4 Laboratory of Social Evolution and Behav- Ior, The Rockefeller University, New York City, USA

## Correction to: Front Zool (2021) 18:46 10.1186/s12983-021-00427-8

Following publication of the original article [1], some errors were found in the online article:

On page 3: in the Fig. 1 caption, the text following (B) and (C) need to be swapped. It should read: ‘(A) *Ecitophya* rove beetle representing the myrmecoid gestalt, (B) *Vatesus* rove beetle as well as (C) *Nymphister* histerid beetle the protective gestalt, (D) *Vatesus* rove beetle larva ….’

On page 8: in the section of ‘Comparisons across ecitophile phenotypic traits’, the sentence ‘This study included six species with such a gestalt (Table 2).’ should be ‘This study included eight species with such a gestalt (Table 2).’

On Page 11, in the section of ‘Comparisons across ecitophile phenotypic traits’, the citation in the sentence ‘Unexpectedly, we found no clear overall effect of CHC host similarity on host aggression (Table 2)’ is incorrect, the citation should be ‘Table 3, so the whole sentence will be: Unexpectedly, we found no clear overall effect of CHC host similarity on host aggression (Table 3).’

On page 14, the word ‘occurred’ was missed in the sentence of ‘For instance, these miniature ecitophiles efficiently avoided contacts with ants by moving away promptly in their typical stop-and-go, zigzag manner, often before physical contact even’, the whole sentence should be: ‘For instance, these miniature ecitophiles efficiently avoided contacts with ants by moving away promptly in their typical stop-and-go, zigzag manner, often before physical contact even occurred.’

The original paper has been updated.

The Publisher apologizes to the authors and the readers for the inconvenience caused by the errors.
