# Supplementary material for: Multiple phenotypic traits as triggers of host attacks towards ant symbionts: body size, morphological gestalt, and chemical mimicry accuracy
Source: Front Zool. 2021 Sep 19;18:46. doi: 10.1186/s12983-021-00427-8 (PMC8451089; doi:10.1186/s12983-021-00427-8)
Supplement: Supplementary file 2 — Additional file 2. The file contains supplemental figures and tables. [file 12983_2021_427_MOESM2_ESM.pdf]

Additional file 2 – Supplemental figures and tables

Multiple phenotypic traits as triggers of host attacks towards ant symbionts: body size, morphological gestalt, and chemical mimicry accuracy

Authors: Christoph von Beeren [a*], Adrian Brückner [b], Philipp Hoenle [a], Brian Ospina-Jara [c], Daniel J.C. Kronauer [d], Nico Blüthgen [a]

Affiliations:

[a] Ecological Networks, Department of Biology, Technical University of Darmstadt, Darmstadt, Germany

[b] Division of Biology and Biological Engineering, California Institute of Technology, Pasadena, USA

[c] Department of Biology, University of Valle, Cali, Colombia

[d] Laboratory of Social Evolution and Behavior, The Rockefeller University, New York City, USA

*Correspondence to:

Christoph von Beeren: cvonbeeren@gmail.com

Content:

Figure S1. Ant behaviors towards ecitophiles and ecitophile dry weight.
Figure S2. CHC profiles of army ant workers.
Figure S3. CHC host resemblance of ecitophiles.
Figure S4. Intraspecific CHC profile differences in the rove beetle *Vatesus* cf. *clypeatus* sp. 2.
Figure S5. Ant aggression towards different types of ecitophile gestalt in relation to CHC host similarity.
Table S1. Univariate data summary of behavioral tests.

Figure S1. Ant behaviors towards ecitophiles and ecitophile dry weight. (a-e) Violin jitter plots showing different ant behaviors towards specimens of different ecitophile genera. The ant behavior *seizing* was uncommon and is not shown here. Note the differing scales on y-axes. (f) Dry weight of ecitophiles in milligram. Species are lumped here within their genera for better data visualization. Raw data contain information for each ecitophile individual (Additional file 1).

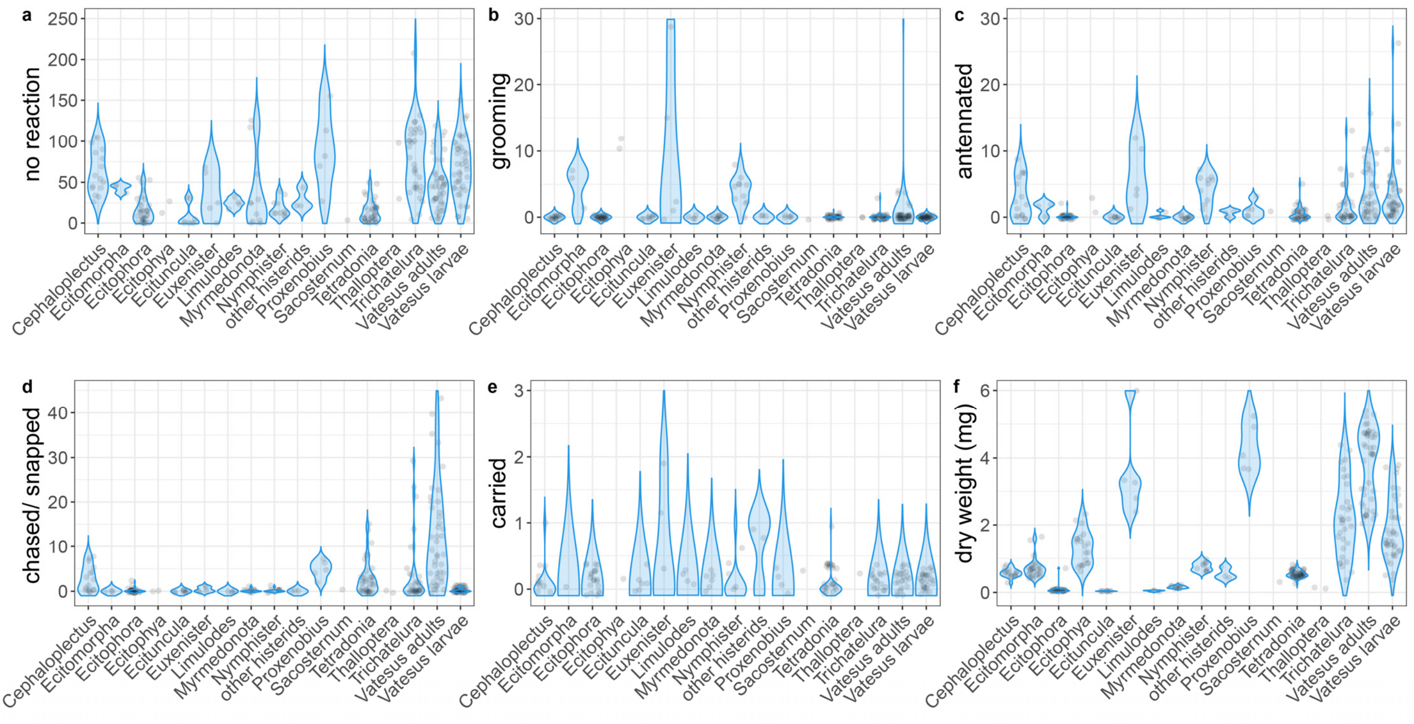

Figure S2. CHC profiles of army ant workers. NMDS plots visualizing the compositional differences of army ant CHC profiles in (a) 2D-space and (b, c) for a subset of data in 3D-space. Data have been rotated in 3D-space for better visualization of compositional species differences. 'Stress' is a quality measure of NMDS.

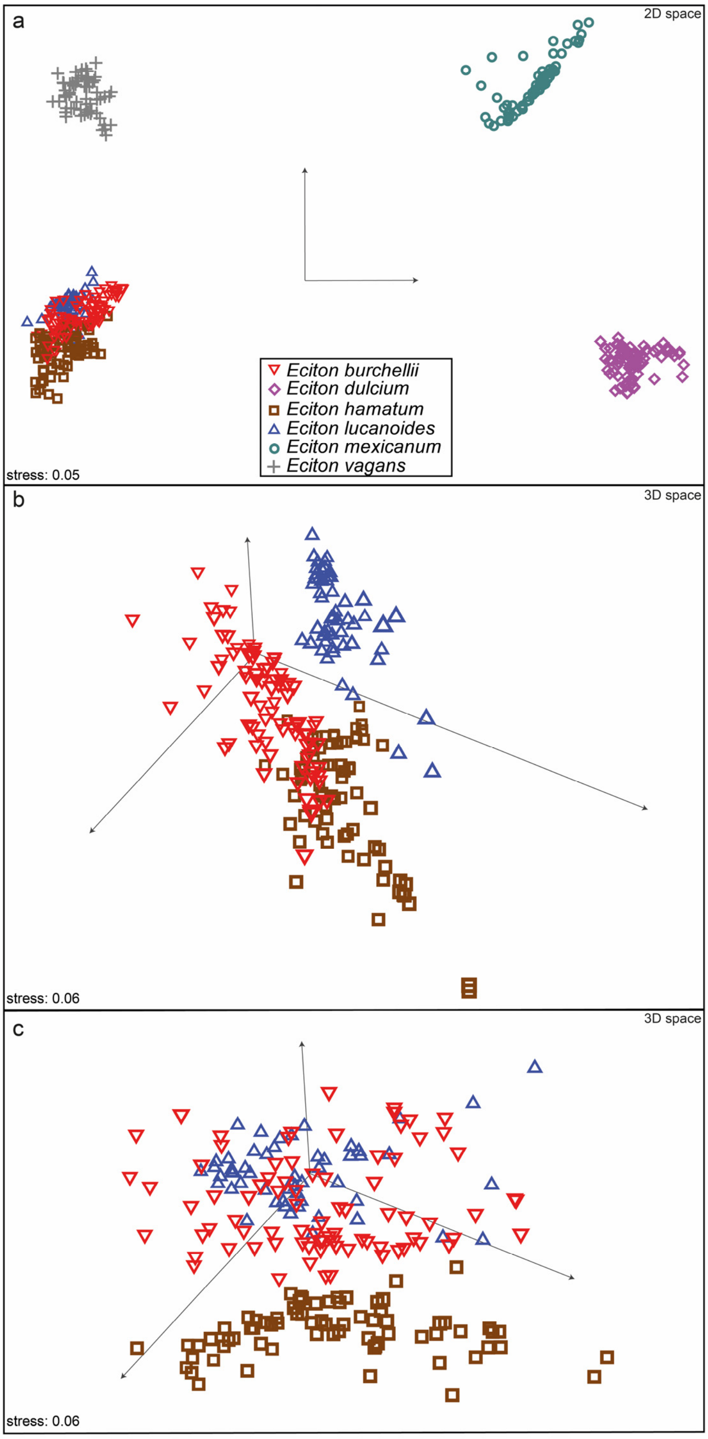

Figure S3. CHC host resemblance of ecitophiles. NMDS plots based on Bray-Curtis similarities visualize CHC profile similarities between host ants and ecitophiles for 14 *Eciton* colonies. Host species and colony IDs are given for each subplot. Asterisks indicate that only NMDS subsets are shown to optimize visualization. Excluded specimens stretched the 2-D space in a way that workers and some ecitophiles were densely clumped. The following species were excluded in NMDS plots: colony EB38D: one *Vatesus* cf. *clypeatus* sp. 2 larva; colony ED36: four *Tetradonia laticeps* specimens and four *Ecitophora pilusola* specimens; colony ED37: eight *T. laticeps* specimens; colony ED38: four *T. laticeps* specimens. 'Stress' is a quality measure of NMDS. Note that we were not able to determine if the two colonies denoted as 'EB38' and 'EB38D' were different *E. burchellii* colonies, as they were collected three weeks apart from each other at the same collection spot (within a radius of < 200m). We treated them as different colonies in the present work. For a likelihood assessment of re-collection events of nomadic army ant colonies at the study site see previous publications [1,2].

References

1. von Beeren C, Maruyama M, Kronauer DJC. Cryptic diversity, high host specificity and reproductive synchronization in army ant-associated *Vatesus* beetles. Mol Ecol. 2016;25:990–1005.

2. Hoenle PO, Blüthgen N, Brückner A, Kronauer DJ, Fiala B, Donoso DA, et al. Species-level predation network uncovers high prey specificity in a Neotropical army ant community. Molecular Ecology. 2019;28:2423–40.

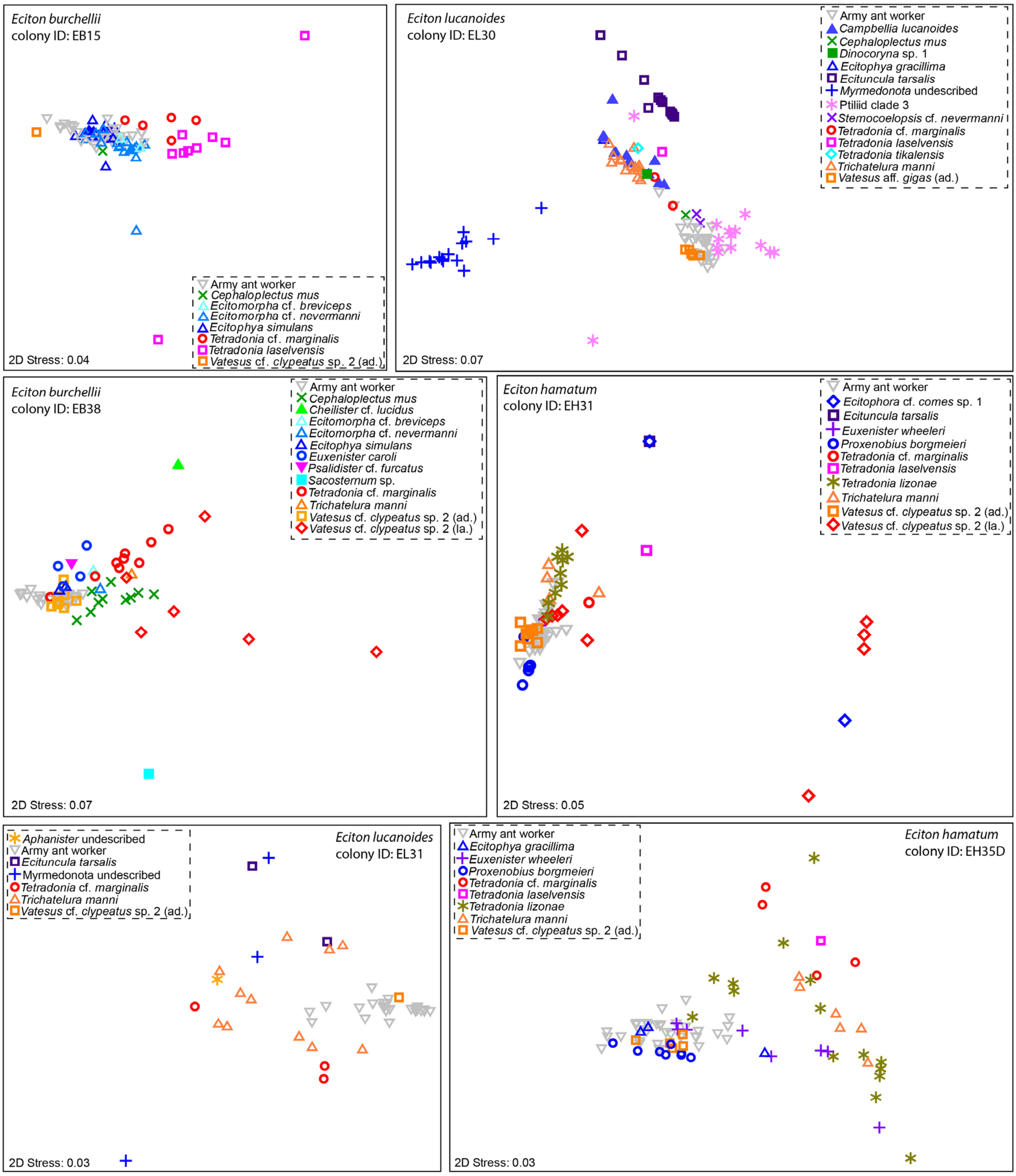
Eciton burchellii
colony ID: EB15
Army ant worker
Cephaloplectus mus
Ecitomorpha cf. breviceps
Ecitomorpha cf. nevermanni
Ecitophya simulans
Tetradonia cf. marginalis
Tetradonia laselvensis
Vatesus cf. clypeatus sp. 2 (ad.)
2D Stress: 0.04
Eciton lucanoides
colony ID: EL30
Army ant worker
Campbellia lucanoides
Cephaloplectus mus
Dinocoryna sp. 1
Ecitophya gracillima
Ecituncula tarsalis
Myrmedonota undescribed
Ptiliid clade 3
Sternocoelopsis cf. nevermanni
Tetradonia cf. marginalis
Tetradonia laselvensis
Tetradonia tikalensis
Trichatelura manni
Vatesus aff. gigas (ad.)
2D Stress: 0.07
Eciton burchellii
colony ID: EB38
Army ant worker
Cephaloplectus mus
Cheilister cf. lucidus
Ecitomorpha cf. breviceps
Ecitomorpha cf. nevermanni
Ecitophya simulans
Euxenister caroli
Psalidister cf. furcatus
Sacosternum sp.
Tetradonia cf. marginalis
Trichatelura manni
Vatesus cf. clypeatus sp. 2 (ad.)
Vatesus cf. clypeatus sp. 2 (la.)
2D Stress: 0.07
Eciton hamatum
colony ID: EH31
Army ant worker
Ecitophora cf. comes sp. 1
Ecituncula tarsalis
Euxenister wheeleri
Proxenobius borgmeieri
Tetradonia cf. marginalis
Tetradonia laselvensis
Tetradonia lizonae
Trichatelura manni
Vatesus cf. clypeatus sp. 2 (ad.)
Vatesus cf. clypeatus sp. 2 (la.)
2D Stress: 0.05
Aphanister undescribed
Army ant worker
Ecituncula tarsalis
Myrmedonota undescribed
Tetradonia cf. marginalis
Trichatelura manni
Vatesus cf. clypeatus sp. 2 (ad.)
Eciton lucanoides
colony ID: EL31
2D Stress: 0.03
Army ant worker
Ecitophya gracillima
Euxenister wheeleri
Proxenobius borgmeieri
Tetradonia cf. marginalis
Tetradonia laselvensis
Tetradonia lizonae
Trichatelura manni
Vatesus cf. clypeatus sp. 2 (ad.)
Eciton hamatum
colony ID: EH35D
2D Stress: 0.03

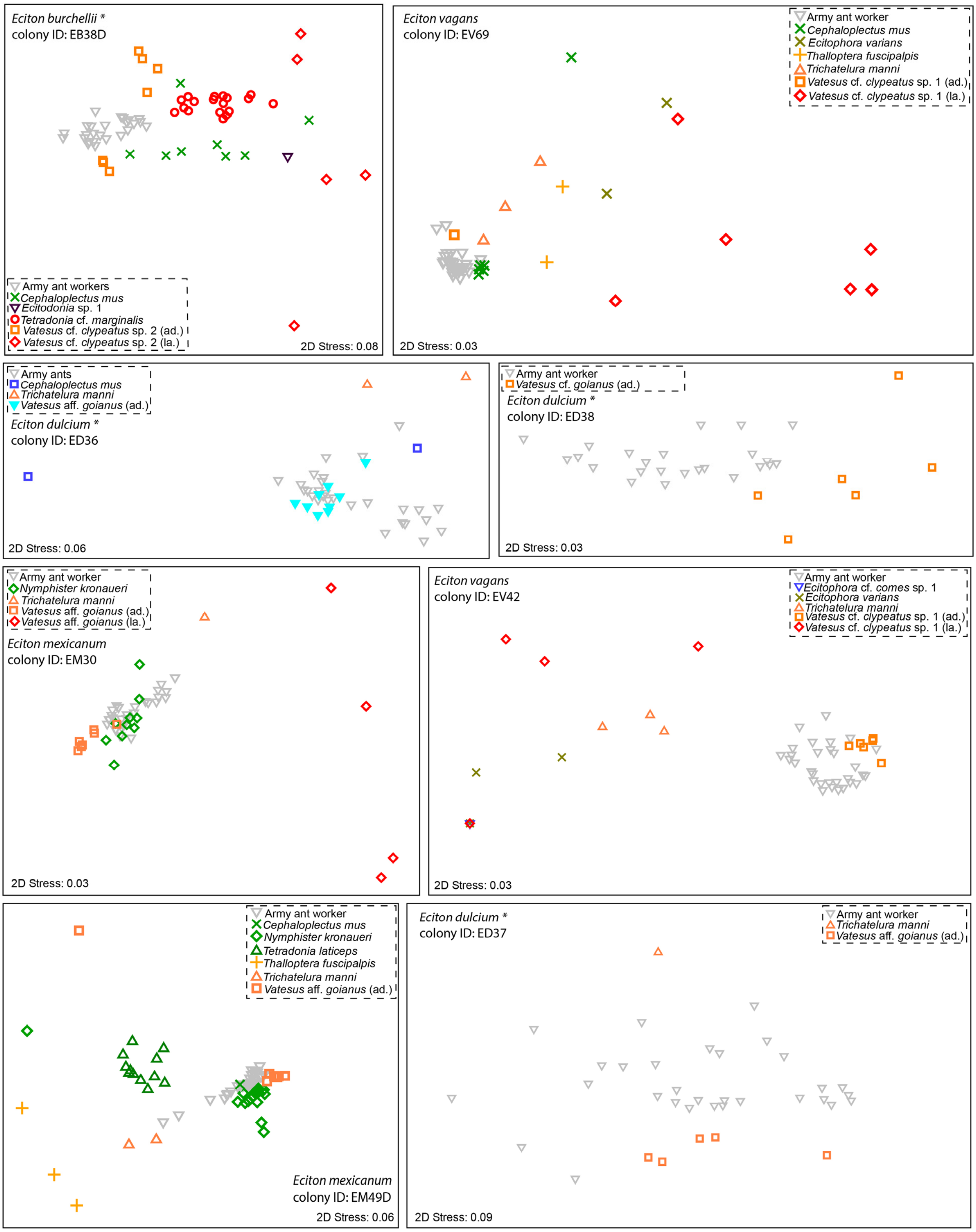

Eciton burchellii *
colony ID: EB38D
Army ant workers
Cephaloplectus mus
Ecitodonia sp. 1
Tetradonia cf. marginalis
Vatesus cf. clypeatus sp. 2 (ad.)
Vatesus cf. clypeatus sp. 2 (la.)
2D Stress: 0.08
Eciton vagans
colony ID: EV69
Army ant worker
Cephaloplectus mus
Ecitophora varians
Thalloptera fuscipalpis
Trichatelura manni
Vatesus cf. clypeatus sp. 1 (ad.)
Vatesus cf. clypeatus sp. 1 (la.)
2D Stress: 0.03
Army ants
Cephaloplectus mus
Trichatelura manni
Vatesus aff. goianus (ad.)
Eciton dulcium *
colony ID: ED36
2D Stress: 0.06
Army ant worker
Vatesus cf. goianus (ad.)
Eciton dulcium *
colony ID: ED38
2D Stress: 0.03
Army ant worker
Nymphister kronaueri
Trichatelura manni
Vatesus aff. goianus (ad.)
Vatesus aff. goianus (la.)
Eciton mexicanum
colony ID: EM30
2D Stress: 0.03
Eciton vagans
colony ID: EV42
Army ant worker
Ecitophora cf. comes sp. 1
Ecitophora varians
Trichatelura manni
Vatesus cf. clypeatus sp. 1 (ad.)
Vatesus cf. clypeatus sp. 1 (la.)
2D Stress: 0.03
Army ant worker
Cephaloplectus mus
Nymphister kronaueri
Tetradonia laticeps
Thalloptera fuscipalpis
Trichatelura manni
Vatesus aff. goianus (ad.)
Eciton mexicanum
colony ID: EM49D
2D Stress: 0.06
Eciton dulcium *
colony ID: ED37
Army ant worker
Trichatelura manni
Vatesus aff. goianus (ad.)
2D Stress: 0.09

Figure S4. Intraspecific CHC profile differences in the rove beetle *Vatesus* cf. *clypeatus* sp. 2. Top: 2D NMDS plots based on Bray-Curtis similarities visualize CHC profile similarities between adults of the rove beetle species *Vatesus* cf. *clypeatus* sp. 2. For better visualization, this graph shows only two of its host ant species, i.e. *E. burchellii* and *E. hamatum*. Bottom: 3D NMDS plot including the third host species *E. lucanoides*. 'Stress' is a quality measure of NMDS.

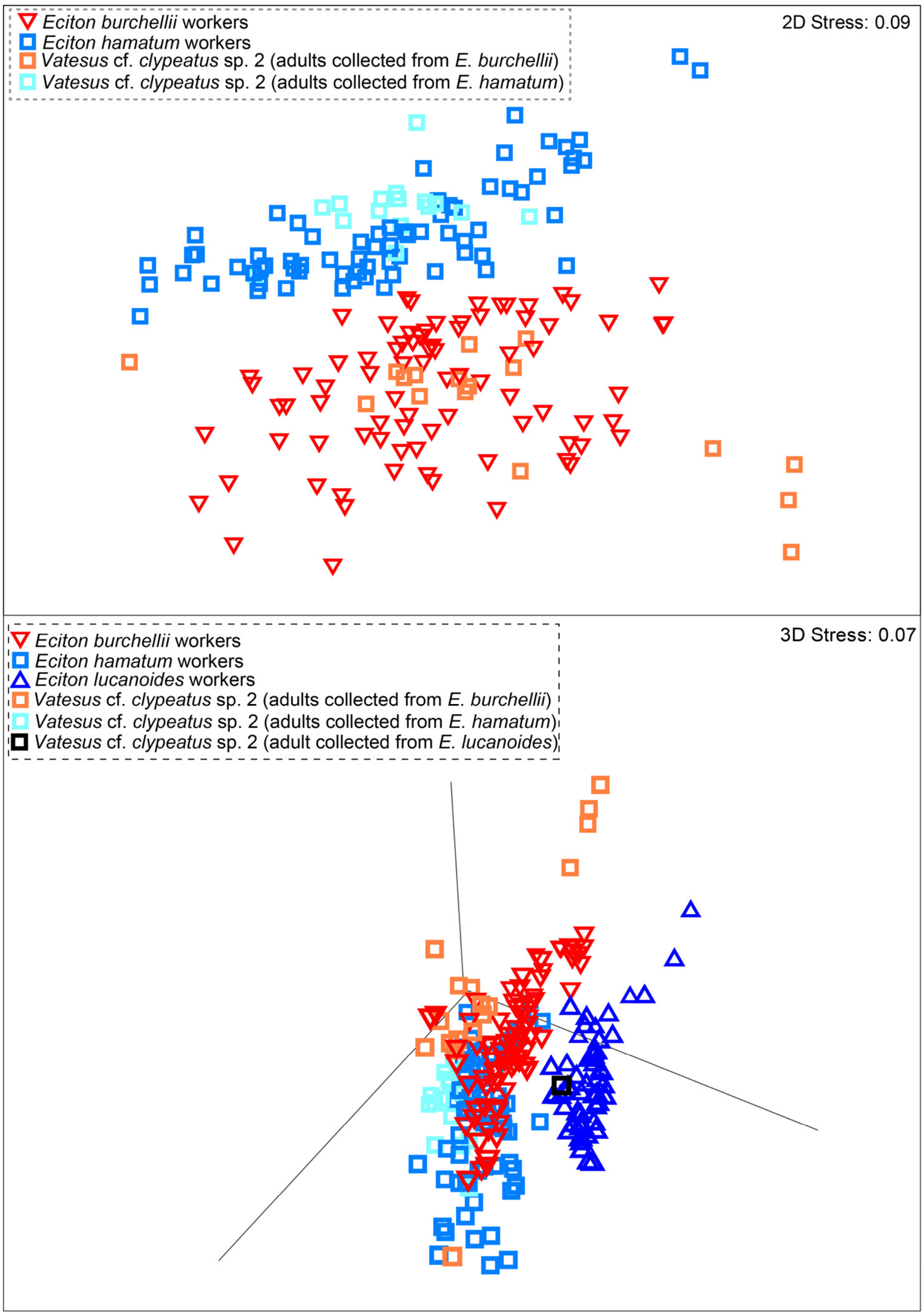

Figure S5. Ant aggression towards different types of ecitophile gestalt in relation to CHC host similarity. The aggression index summarizes the proportion of aggressive ant responses towards ecitophiles relative to the total number of contacts. CHC host similarity is given as Bray-Curtis similarity of an ecitophile to the centroid of its host colony. Size of data points correspond to dry weight as given in the figure legend. Note that the scale in CHC host similarity can be different in subplots.

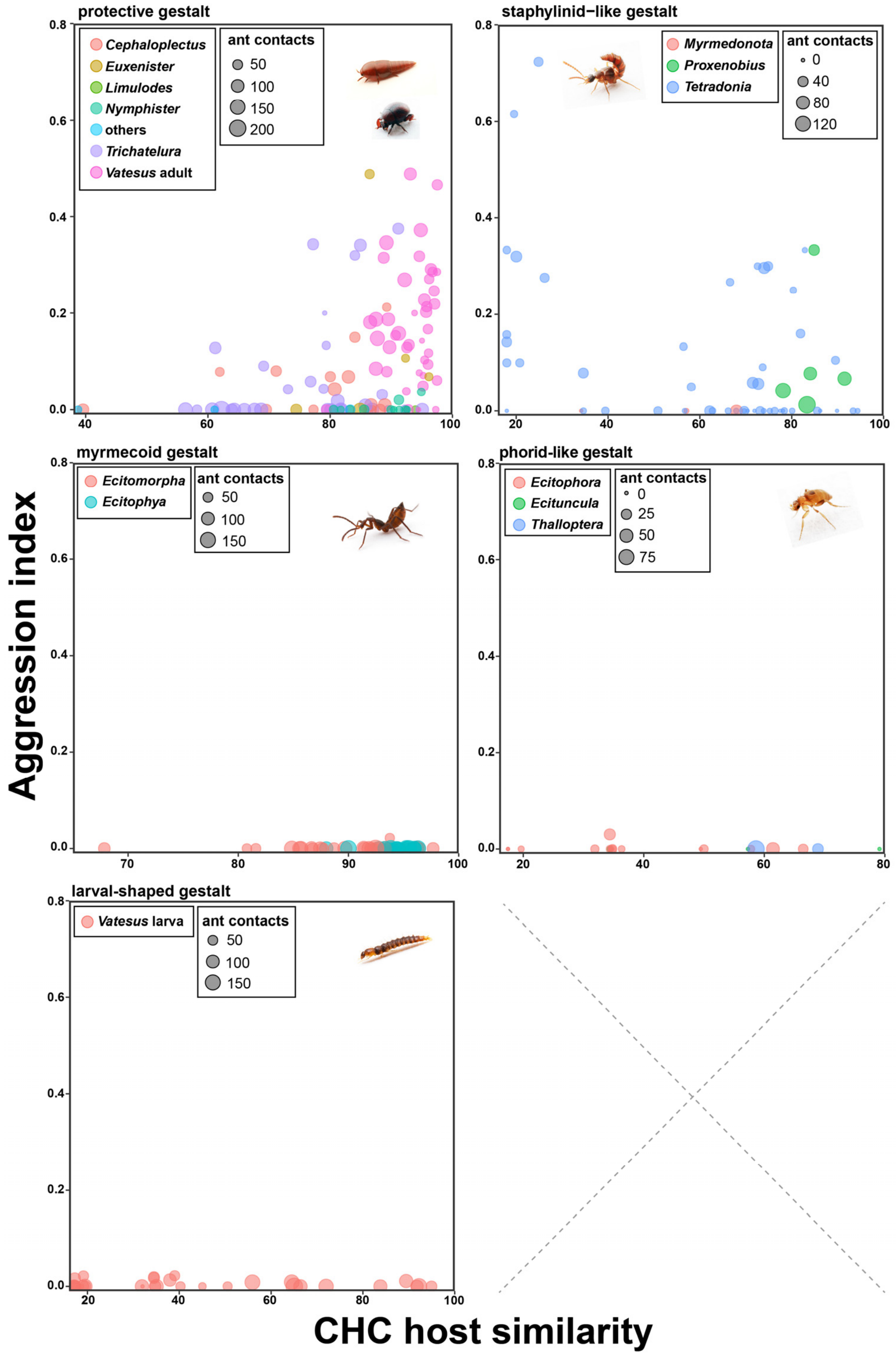

Table S1. Univariate data summary of behavioral tests. Given are the number of tested specimens per ecitophile species (sample size) and the means ± standard deviations of ant behaviors towards ecitophiles.

Order	Family	Ecitophile species	Sample size	no reaction	grooming	antennating	chasing/snapping	stinging	carrying	seizing
Coleoptera	Histeridae	*Aphanister* sp. 1	1	21	0	0	0	0	0	0
		Cheilister cf. *lucidulus*	1	21	0	1	0	0	1	0
		Euxenister caroli	4	37 ± 33	12 ± 13	7 ± 4	0 ± 1	2 ± 3	1 ± 1	4 ± 7
		Euxenister wheeleri	1	25	1	1	1	0	0	1
		Nymphister kronaueri	11	18 ± 11	4 ± 2	4 ± 2	0 ± 0	0 ± 0	0 ± 0	0 ± 0
		Psalidister cf. *furcatus*	1	44	0	1	0	0	1	0
	Hydrophilidae	*Sacosternum* aff. *lebbinorum*	1	3	0	1	0	0	0	0
	Ptiliidae	*Cephaloplectus mus*	16	64 ± 27	0 ± 0	3 ± 3	3 ± 3	0 ± 0	0 ± 0	0 ± 0
		Limulodes sp. 2	2	25 ± 2	0 ± 0	1 ± 1	0 ± 0	0 ± 0	0 ± 0	0 ± 0
		Limulodes sp. 3	2	25 ± 11	0 ± 0	0 ± 0	0 ± 0	0 ± 0	0 ± 0	0 ± 0
	Staphylinidae	*Ecitomorpha* cf. *breviceps*	2	41 ± 6	4 ± 4	2 ± 0	0 ± 0	1 ± 1	0 ± 0	0 ± 0
		Ecitomorpha cf. *nevermanni*	1	47	7	0	0	0	0	0
		Ecitophya simulans	2	20 ± 11	11 ± 1	2 ± 1	0 ± 0	0 ± 0	0 ± 0	0 ± 0
		Ecituncula tarsalis	8	9 ± 14	0 ± 0	0 ± 0	0 ± 0	0 ± 0	0 ± 0	0 ± 0
		Myrmedonota sp. 1	11	36 ± 46	0 ± 0	0 ± 0	0 ± 0	0 ± 0	0 ± 0	0 ± 0
		Proxenobius borgmeieri	5	89 ± 48	0 ± 0	1 ± 1	5 ± 2	2 ± 3	0 ± 0	0 ± 0
		aff. *Tetradonia* sp. 1	1	20	0	0	5	0	1	0
		Tetradonia cf. *marginalis*	14	13 ± 13	0 ± 0	1 ± 1	1 ± 1	0 ± 0	0 ± 0	0 ± 0
		Tetradonia laselvensis	1	48	0	0	3	0	0	0
		Tetradonia laticeps	12	17 ± 11	0 ± 0	0 ± 1	5 ± 5	0 ± 1	0 ± 0	0 ± 1
		Tetradonia lizonae	9	10 ± 12	0 ± 0	1 ± 2	3 ± 4	1 ± 1	0 ± 0	0 ± 0
		Adult *Vatesus* aff. *goianus*	14	32 ± 16	0 ± 0	1 ± 1	9 ± 9	0 ± 1	0 ± 0	0 ± 0
		Larval *Vatesus* aff. *goianus*	7	72 ± 25	0 ± 0	2 ± 3	1 ± 1	0 ± 0	0 ± 0	0 ± 0
		Adult *Vatesus* cf. *clypeatus* sp. 1	7	100 ± 14	0 ± 0	5 ± 3	16 ± 5	3 ± 2	0 ± 0	0 ± 0
		Larval *Vatesus* cf. *clypeatus* sp. 1	14	54 ± 26	0 ± 0	2 ± 2	0 ± 0	0 ± 0	0 ± 0	0 ± 0
		Adult *Vatesus* cf. *clypeatus* sp. 2	20	41 ± 23	2 ± 7	6 ± 4	14 ± 14	3 ± 4	0 ± 0	0 ± 0
		Larval *Vatesus* cf. *clypeatus* sp. 2	21	73 ± 41	0 ± 0	5 ±6	0 ± 1	0 ± 1	0 ± 0	0 ± 0
Diptera	Phoridae	*Ecitophora bruchi*	6	14 ± 7	0 ± 0	0 ± 0	0 ± 0	0 ± 0	0 ± 0	0 ± 0
		Ecitophora cf. *comes* sp. 1	9	15 ± 18	0 ± 0	0 ± 1	0 ± 1	0 ± 0	0 ± 0	0 ± 0
		Ecitophora pilosula	10	22 ± 20	0 ± 0	0 ± 0	0 ± 0	0 ± 0	0 ± 0	0 ± 0
		Ecitophora varians	5	7 ± 7	0 ± 0	0 ± 0	0 ± 0	0 ± 0	0 ± 0	0 ± 0
		Thalloptera fuscipalpis	2	64 ± 48	0 ± 0	0 ± 0	0 ± 0	0 ± 0	0 ± 0	0 ± 0
Thysanura	Nicoletiidae	*Trichatelura manni*	32	79 ± 43	0 ± 1	2 ± 3	4 ± 7	0 ± 1	0 ± 0	0 ± 0
