# Supplementary material for: Multiple phenotypic traits as triggers of host attacks towards ant symbionts: body size, morphological gestalt, and chemical mimicry accuracy
Source: Front Zool. 2021 Sep 19;18:46. doi: 10.1186/s12983-021-00427-8 (PMC8451089; doi:10.1186/s12983-021-00427-8)
Supplement: Supplementary file 3 — Additional file 3. Supplemental experiments addressing the influence of CHC concentration on host aggression and the acquisition of CHCs by ecitophiles. [file 12983_2021_427_MOESM3_ESM.pdf]

Additional file 3 - Supplemental experiments

Multiple phenotypic traits as triggers of host attacks towards ant symbionts: body size, morphological gestalt, and chemical mimicry accuracy

Authors: Christoph von Beeren [a*], Adrian Brückner [b], Philipp Hoenle [a], Brian Ospina Jara [c], Daniel J.C. Kronauer [d], Nico Blüthgen [a]

Affiliations:

[a] Ecological Networks, Department of Biology, Technical University of Darmstadt, Darmstadt, Germany

[b] Division of Biology and Biological Engineering, California Institute of Technology, Pasadena, USA

[c] Department of Biology, University of Valle, Cali, Colombia

[d] Laboratory of Social Evolution and Behavior, The Rockefeller University, New York City, USA

*Correspondence to:

Christoph von Beeren: cvonbeeren@gmail.com

Content:

Supplemental experiment 1: CHC concentrations of ecitophiles

Supplemental experiment 2: Label transfer from workers to ecitophiles

Supplemental experiment 1: CHC concentrations of ecitophiles

Mimicking host CHC profiles in chemical composition is a widespread phenomenon among social insect guests [1,2]. This chemical mimicry (*sensu* [3]) of host recognition cues is generally believed to facilitate peaceful host-myrmecophile interactions [1]. Whether the amount/concentration of these mimetic compounds besides their composition additionally plays a role in facilitating host-symbiont interactions remains largely unclear [4]. Further, there are several lines of evidence suggesting that chemical hiding (*sensu* [3]) plays an important role in avoiding rejection by social insect workers [5–9]; see also [9–11] for chemical insignificance and chemical transparency). Here social insect guests carry no or only little amounts of CHCs which is expected to hamper their recognition as intruders [1]. For instance, Cini et al. (2009) suggested a quantitative threshold for nestmate recognition in the paper wasp *Polistes dominulus* [8]. In line with these findings, we expected ecitophiles with no detectable CHCs, or minimal CHC concentrations, to receive less aggression because of staying undetected by the army ants' chemical recognition system.

However, there is a methodological problem when studying CHC concentrations between hosts and social insect symbionts: specimens can have vastly different body sizes. The detected CHC amounts of a specimen must therefore be standardized for body size (e.g., [4,7]), best for surface area. One approach to do this is to use the dry weight as estimator for surface area (weight and volume are linearly related; for details see [12]). However, this surface area approximation has its limitations. When studying specimens of vastly different body shapes and/or different levels of cuticular plating it only inaccurately characterizes the relative surface areas of different insect species [12]. As an example, we analyzed the surface area via µCT scans of two ecitophilous specimens in a preliminary study (Fig. S1 of this file). A specimen of the limuloid silverfish *Trichatelura manni* had approximately the same surface area as a specimen of the limuloid rove beetle *Vatesus* cf. *clypeatus* sp. 2 (Fig. S1 of this file). Due to its thicker cuticular shielding, however, the rove beetle was 2.68 times heavier than the silverfish (Fig. S1 of this file), demonstrating the inaccuracy in estimating surface areas via the dry weight when comparing vastly different species. We thus decided to treat the herein presented comparison of CHC concentrations between ecitophile species as a preliminary study. Being aware of the limitations, we decided to still present the data on CHC concentrations in this supplement, because we consider these data meaningful when considering specimens showing signs of chemical hiding, that is those specimens carrying no to little CHC concentrations.

Methods - CHC concentrations

To study CHC concentrations we first calculated CHC amounts [in ng] using the internal standard octadecane [13]. To account for body size differences between specimens we standardized CHC amounts in a second step using the dry weight as estimator for surface area. We used the dry weight of each specimen to the power of two-third [$mg^{2/3}$] to account for the faster increase of volume compared to surface area (for more details see [4,12]).

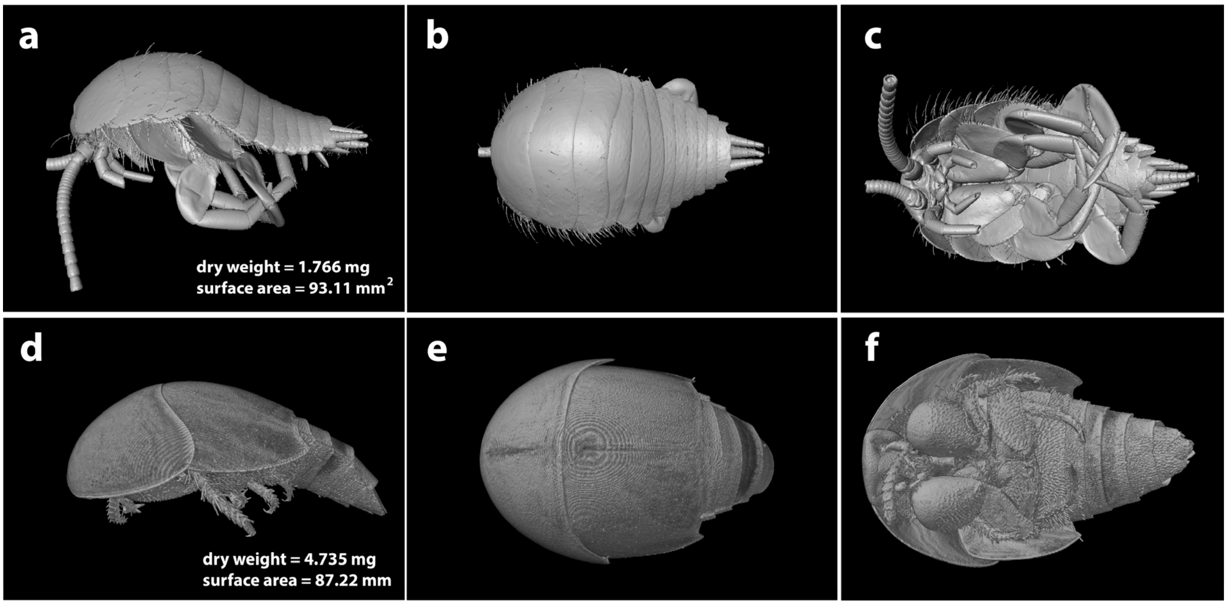

Figure S1. Micro-CT scans of two ecitophiles. (a) Lateral, (b) dorsal and (c) ventral view of a µCT scan of the silverfish species *Trichatelura manni.* (d) Lateral, (e) dorsal and (f) ventral view of a µCT scan of the rove beetle species *Vatesus* cf. *clypeatus* sp. 2. Synchrotron-generated X-ray microtomography (SRµCT) were conducted at the ANKA of the Karlsruhe Institute of Technology as part of the NOVA project in cooperation with Sebastian Schmelzle and Michael Heethoff. 3-D surface models were created with the software AMIRA. Details are given in [12,14].

We expected CHC concentrations [ng/ $mg^{2/3}$] on workers to be relatively constant due to the frequent exchange of CHCs among ant nestmates [15]. Concordantly, we expected that larger workers carry higher amounts of CHCs due to their increased surface area. Indeed, our data indicated that the dry weight is a good predictor of the ants' surface area as we found a positive linear relationship between dry weight and CHC amount in *Eciton* workers (linear model: N = 396, F-value: 643, $p < 0.001$; Fig. S2 of this file). We tested for differences in CHC concentrations between army ant species by running a linear mixed-effects model (lmer) using the log-transformed CHC concentration (log (CHC conc. +1)) as response variable and army ant species as explanatory variable. Colony was set as random factor. The same model design was used when testing for differences between ecitophiles, except that ecitophile species was used as explanatory variable. The inspection of the models residual distributions detected no significant problem, which we examined by using the function plotResiduals() as implemented by the package 'DHARMa' [89]. We tested both models by running a type-III Wald chi square analysis-of-variance. Note that sample sizes partly differ between the analyses of BC similarity and CHC concentration. This is because specimens without any detectable CHCs were excluded from compositional data analysis. In addition, we did not measure the dry weight for some ecitophiles because specimens were either lost or already deposited at museum collections.

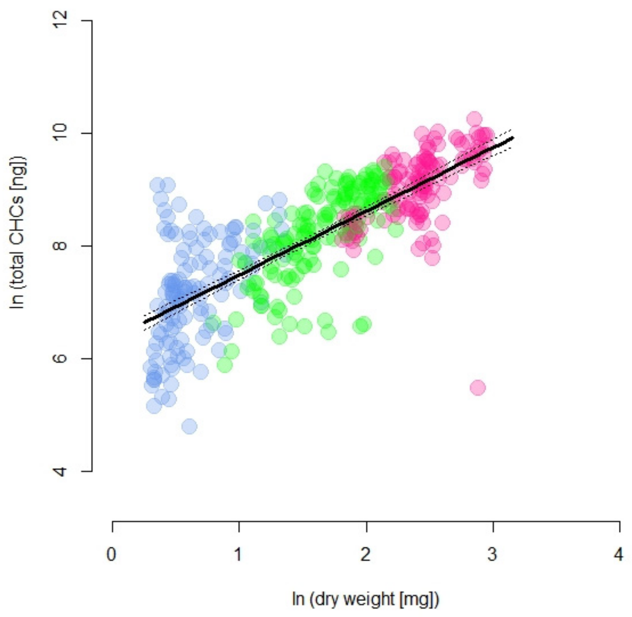

Figure S2. Relationship between dry weight and ant worker CHC amount. Included are six *Eciton* species. CHC amount is plotted against animal dry weight for *Eciton* minors (blue circles, N = 128), *Eciton* intermediates (green circles, N = 139), and *Eciton* majors (pink circles, N = 129). We calculated a linear model with dry weight as independent and CHC amount as dependent variable. We log-transformed both variables to follow a log-normal distribution: log (dry weight + 1) and log (CHC amount + 1). Solid black line shows the linear line of best fit and dashed line the 95% confidence interval.

Results and Discussion - CHC concentrations

The CHC concentrations in army ant workers were quite variable ranging from a minimum of 36 $ng/mg^{2/3}$ to a maximum of 7921 $ng/mg^{2/3}$ (median CHC concentration = 1628 $ng/mg^{2/3}$, N = 396 workers; Fig. S3 of this file). We found a trend indicating that CHC concentrations differed between *Eciton* species (lmer: $\chi^2 = 10.89$, df = 5, $p = 0.053$; Fig. S3 of this file).

Like workers, CHC concentrations of ecitophiles were variable and they clearly differed between species (lmer: $\chi^2 = 841$, df = 35, $p < 0.001$). Highest CHC concentrations were detected in species of the staphylinid genera *Vatesus* (median of adults = 3764 $ng/mg^{2/3}$, N = 46), *Proxenobius* (median = 1520 $ng/mg^{2/3}$, N =13), and *Campbellia* (median = 1350 $ng/mg^{2/3}$, N =13) (Fig. S4a). Low to no detectable CHC concentrations were found in *Vatesus* larvae (median = 6 $ng/mg^{2/3}$) and all species of phorid flies (all phorids: median = 0 $ng/mg^{2/3}$) (Fig. S4a of this file).

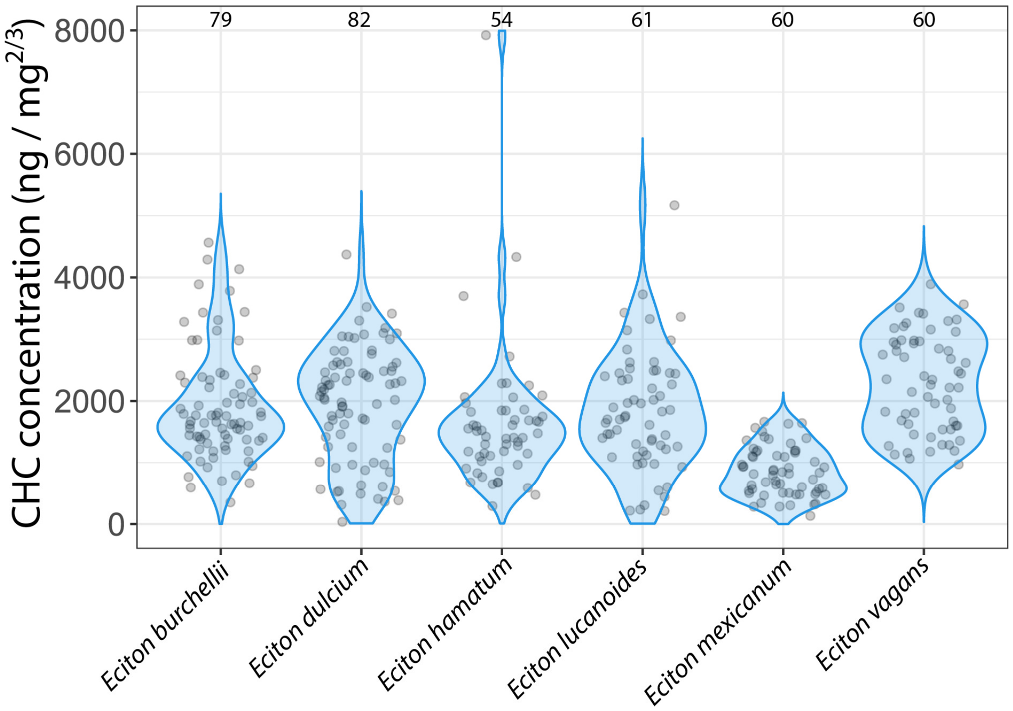

Figure S3. CHC concentrations of 396 Eciton workers. Violin jitter plots showing CHC concentrations in *Eciton* workers of the six studied species. Sample sizes are given above violin plots. We found a trend indicating that CHC concentrations between *Eciton* species might differ (lmer: $\chi^2 = 10.89$, df = 5, p = 0.053).

We found a positive correlation between the aggression index and the CHC concentrations (Spearman rank correlation: $\rho = 0.469$, $p < 0.001$; Fig. S4b of this file) as well as between the sum of aggressive behaviors against ecitophiles and their CHC concentrations (Spearman rank correlation: $\rho = 0.479$, $p < 0.001$). As expected, specimens having extremely low CHC concentrations (< 100 ng/mg$^{2/3}$) were rarely attacked (14 out of 85 ecitophiles attacked once; Fig. S4a of this file). Ecitophiles with low CHC concentrations included a diverse spectrum of taxa: one specimen of the hydrophilid species *Sacosternum* aff. *lebbinorum*, 30 specimens of the phorid fly genera *Ecitophora* and *Ecituncula*, three specimens of the limuloid ptiliid genus *Limulodes*, one specimen of the myrmecoid rove beetle genus *Ecitophya*, nine specimens of the rove beetle genus *Myrmedonota*, one *Tetradonia laselvensis* specimen, four specimens of the limuloid silverfish *Trichatelura manni*, and 37 specimens of *Vatesus* larvae (Additional file 1). Except of *Vatesus* larvae, *T. manni* silverfish, and *Ecitophya* and *Tetradonia* beetles, all remaining individuals were also small and had a dry weight of less than 0.307 mg (Additional file 1). In contrast, ecitophiles with high CHC concentrations (> 3000 ng/mg$^{2/3}$) were attacked more frequently (33 out of 38 ecitophiles attacked at least once; 501 attacks in total; Additional file 1). This category included three specimens of the limuloid ptiliid beetle *Cephaloplectus mus*, one specimen of the phorid fly *Ecituncula tarsalis*, one specimen of the staphylinid-like rove beetle *Proxenobius borgmeieri*, one specimen of the staphylinid-like rove beetle *Tetradonia* cf. *marginalis*, two specimens of the limuloid silverfish species *Trichatelura manni*, and 30 adult specimens of the rove beetle genera *Vatesus* (Additional file 1).

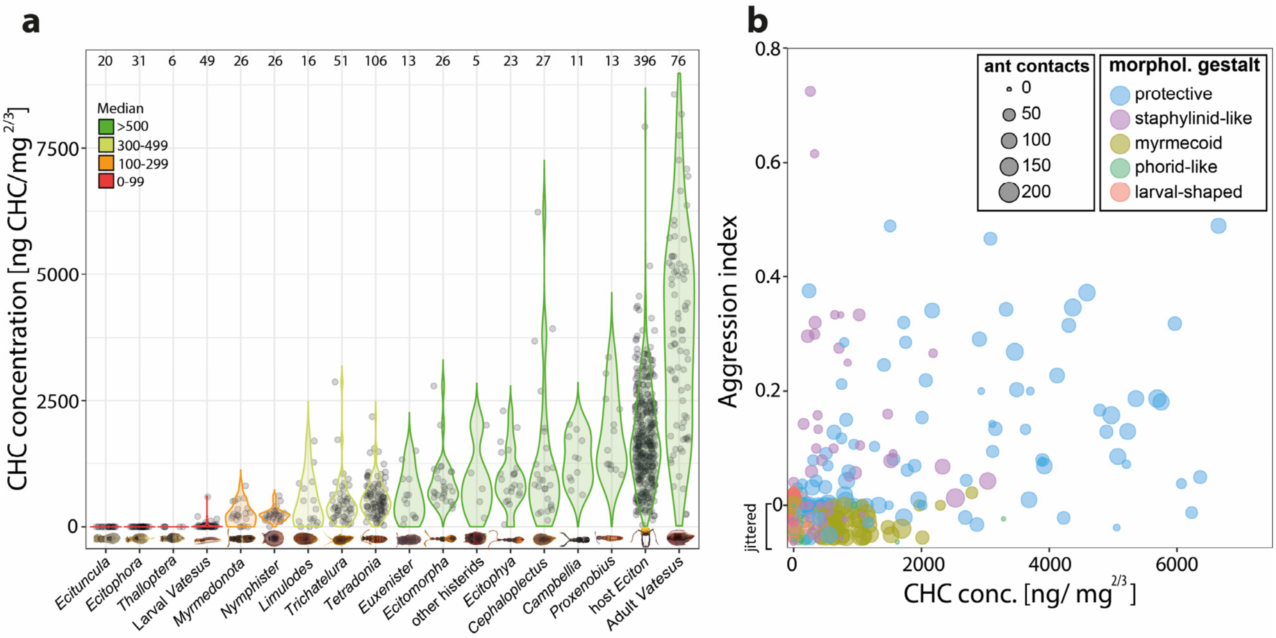

Figure S4. CHC concentrations in ecitophiles and host aggression in relation to CHC concentration. (a) Violin jitter plot showing the CHC concentrations of ecitophiles. For better data visualization, species are lumped within their genera. Raw data includes information at the species level (Additional file 1). Sample sizes are given above violin plots, which are ordered according to the groups' medians. The category 'other histerids' include the species *Aphanister* sp. 1, *Cheilister* cf. *lucidulus*, *Psalidister furcatus*, and *Sternocoelopsis* cf. *nevermanni*. Images are not to scale. (b) Ant aggression towards ecitophiles in relation to their CHC concentrations. The aggression index gives the sum of aggressive interactions (attempt of *chasing*, *snapping*, *stinging,* and *seizing*) divided through the total number of contacts. Colors depict the morphological gestalt of ecitophiles, and size of data points the number of contacts.

We interpret these results as evidence for chemical hiding in certain ecitophiles. Especially *Vatesus* larvae are interesting in this context, because specimens were generally relatively large (dry weight ± SD: mean = 1.80 mg ± 0.85 mg, range = 0.37-3.79 mg, N = 49) but carried little CHC amounts. The low frequency of host attacks against *Vatesus* larvae might thus be partly explained by a lack of host ants to recognize these guests via olfactory cues. However, as discussed in the main article, other traits such as the long macrosetae might also be responsible for the host tolerance of these intruders and we cannot pinpoint the contribution of each trait.

As we found solely CHCs on ecitophiles that were also present in host chemical profiles, we wondered why high concentrations of these mimetic CHCs goes along with a higher probability of being aggressed. One possibility could be that extremely high concentrations increase the likelihood of being recognized as intruder because concentrations falling out of the host's concentration range might be detected as 'invalid' to the ants. However, it is difficult to explain how an ecitophile can even reach CHC concentrations higher than that of their host ants as we expect CHCs to be mainly acquired from the ants (see Supplemental experiment 2 below). We have no evidence-based explanation for this pattern and further studies using manipulative experiments are needed for verification.

Ecitophiles having high CHC concentrations were mostly of the protective gestalt (Fig. S4b), and most of the specimens were *Vatesus* adults. We can think of one possible explanation of why these ecitophiles were aggressed by host ants, although showing high accuracy in resembling the chemical host profile – a mismatch in body shape and/or cuticular sculpturing. High CHC concentrations together with high accuracy in mimicking the composition of CHC profiles might trigger some ants to interact more intensively with their opponent as the olfactory cues suggest the opponent is a nestmate worker. Living in a social insect society implies to have frequent contact with nestmates, including reciprocal antennation, grooming, and, in some species, mouth-to-mouth feeding [1,4,113,114]. A myrmecophile mimicking the host's chemical profile uses a worker ant as a model, which resembles an entity of potential interest to other ant workers [32]. This implies that mimicking an ant's smell with high accuracy should stimulate some worker ants to antennate or groom the mimic, and this is what we frequently observed in myrmecoid beetles, where interactions remained calm and peaceful (Additional file 6). The latter was not the case in species with a limuloid and tortoise-like gestalt, where initial inspection by antennation often led to ant aggression. In other words, when the opponent's body shape and/or cuticular sculpturing does not fit to the morphological gestalt expected by an ant worker during tactile inspection, it might be recognized as a gestalt mismatch, thus triggering an aggressive response.

Supplemental experiment 2: Label transfer from workers to ecitophiles

The most common strategy of myrmecophiles to acquire mimetic CHCs is arguably through physical contact with host ants [1,16]. We thus expected that acquired chemical mimicry (*sensu* [3]) is a common strategy among ecitophiles. To assess the degree of CHC acquisition, we studied the transfer of a labeled CHC from the cuticle of ant workers to ecitophiles. Unfortunately, most of the non-myrmecophilous control isopods in these experiments died within 24h and thus the degree of active label acquisition in ecitophiles from workers cannot be distinguished from a passive label transfer under the laboratory condition. In other words, the label might have spread throughout the laboratory nest so that CHCs might have been acquired from other materials than from ant workers (see also [16,17]). We still decided to provide these data as some insights can be gained when comparing ecitophile species with each other.

Methods - label transfer experiment

We evaluated the transfer of a labeled CHC from the cuticle of ant workers to ecitophiles in one *E. burchellii* colony, one *E. dulcium* colonies, one *E. hamatum* colony and one *E. mexicanum* colony. We set up laboratory nests as described in the main article, except that we additionally added at least 10 isopods per colony as control animals. Isopods were collected haphazardly from the forest floor. They were not expected to search close contact to army ants and therefore served as controls to measure the background noise of label transfer (see also [16,17]). We treated approx. 50 intermediate workers with the stable isotope-labeled hydrocarbon tetracosane-d50 (kindly provided by S. Schulz, TU Braunschweig). We used this label because it has similar properties as natural ant CHCs and because the label was easily recognizable in GC-MS runs by its mass spectrum (molecule ion $M^+ = 389$). For labelling ants, we evaporated a saturated tetracosane-d50 - hexane solution in clean 50 ml glass vials so that the label fully covered the bottom and side walls of the vial as a crystalline film. We then added approx. 50 intermediate workers, shook the vial gently and left the workers in the vial for approx. 30 min. Ant workers did not visibly suffer from this treatment. We verified the success of label transfer in one *Eciton hamatum* colony (median label concentration in intermediate workers: 1320 ng/ $mg^{2/3}$; range: 582-4100 ng/ $mg^{2/3}$, N = 8).

Labeled workers together with approx. 300-400 non-labeled nestmates and 50-100 brood items, all associated ecitophiles, and isopods were then kept together in laboratory nests for 24h. Subsequently we extracted CHCs of more than 20 army ant workers including specimens of each size class (Additional file 1) as described in the main text of the article. We collected haphazardly from the laboratory nests, meaning the extracted specimens could include previously labelled workers. We also extracted all ecitophiles and isopods. As most control isopods died (see results) we were not able to test for differences between control animals and myrmecophiles in CHC label. Nonetheless, we tested for differences in label concentrations between ecitophiles using a linear mixed-effects model (lmer) with log-transformed label concentration (log (CHC conc. +1)) as response variable and ecitophile species as explanatory variable. Colony was set as random factor. Overdispersion and variance homogeneity was checked as described above. We used a type-III Wald chi square analysis-of-variance for statistical testing.

Results and discussion - label transfer experiment

Twenty-four hours after labelling 50 *Eciton* workers per laboratory nest, we detected the CHC label tetracosane-d50 on 95 of 104 extracted workers in variable label concentrations (median across colonies = 7 $ng/mg^{2/3}$, range: 0-124 $ng/mg^{2/3}$; Fig. S5 of this file). This suggests that the label was transferred from the initially labelled workers to most workers of the laboratory nests. Workers of the four laboratory colonies showed vastly different label concentrations (linear model: $F = 80.51$, $p < 0.001$, $N = 104$ workers), with highest concentrations in the *E. burchellii* colony (median concentration = 32.18 $ng/mg^{2/3}$) and lowest ones in the *E. dulcium* colony (median concentration = 1.72 $ng/mg^{2/3}$)(Fig. S5 of this file). We assume that these vast concentration differences might have arisen due to varying efficiencies in initially transferring the label to the workers so that this procedure needs to be better standardized for future work.

Only nine control isopods survived the 24h in laboratory colonies, while most were found dead and partly dismembered, suggesting that *Eciton* workers killed them. Hence, we were not able to reliably quantify the background transfer of the chemical label to specimens in the experimental setups. Of the nine isopods, 5 specimens carried the label, mostly in low concentrations (concentration range: 0-22 $ng/mg^{2/3}$, median = 1 $ng/mg^{2/3}$; Fig S5 of this file). Due to the death of control animals, the degree of active label acquisition in ecitophiles cannot be distinguished from a passive label transfer.

Some insights can still be gained when comparing ecitophile species with each other, because species differed in label concentrations (lmer: $\chi^2 = 82.13$, $df = 14$, $p < 0.001$; Fig. S5 of this file). Overall, we detected the chemical label on 101 out of 147 myrmecophile specimens, which included specimens of all species except of the phorid flies *Ecitophora* cf. *comes* sp. 1 (N = 1) and *Thalloptera fulcipalpis* (N = 5), as well as the rove beetle *Tetradonia laselvensis* (N = 1). Relatively high label concentrations were detected in the rove beetle genera *Ecitophya* (label median across colonies = 11 $ng/mg^{2/3}$, N = 3) and *Vatesus* (label median of adults across colonies = 5 $ng/mg^{2/3}$, N = 24), but even in these two genera we found specimens with no detectable label (*Vatesus* adults = 3 specimens; *Ecitophya* = 1 specimen). Specimens of all other genera mostly had relatively low label concentrations, with the exception of one *Cephaloplectus mus* specimen (label concentration = 31 $ng/mg^{2/3}$) and one *Vatesus* cf. *clypeatus* sp. 2 larva (label concentration = 935 $ng/mg^{2/3}$)(Fig. S5 of this file).

Irrespective of whether the label was transferred passively in the laboratory nest or whether it was actively acquired by ecitophiles from host ants, a large proportion of ecitophiles carried the label on their cuticle. This demonstrated that CHC transfer from host ants to ecitophiles was taking place. Such label transfer between myrmecophiles and host ants had been previously demonstrated in *Leptogenys*-associated myrmecophiles [16,17] and we expected it to be the most common strategy of acquiring mimetic CHCs in ecitophiles. This is because many ecitophiles actively seek contact to host ants and intensively rub their legs on the ants or lick them [4,18] (see Additional files 6-8,10). For instance, Rettenmeyer and Akre speculated that the dense clusters of setae on the inner surface of each tibia in the histerid *Euxenister caroli* represent 'tibial brushes' which facilitate the transfer of host cuticular compounds [18,19](Additional file 10). Another line of evidence for the acquisition of mimetic cues from host ants was the observation that multi-host guests most closely mimicked the chemical profile of those army ant species from which they were collected from (see Fig. 2c, e, f of the main text). It seems unlikely to us that an ecitophile acquired the relevant biochemical pathways during its evolution to *de novo* biosynthesize the species-specific CHC profiles of different host species.

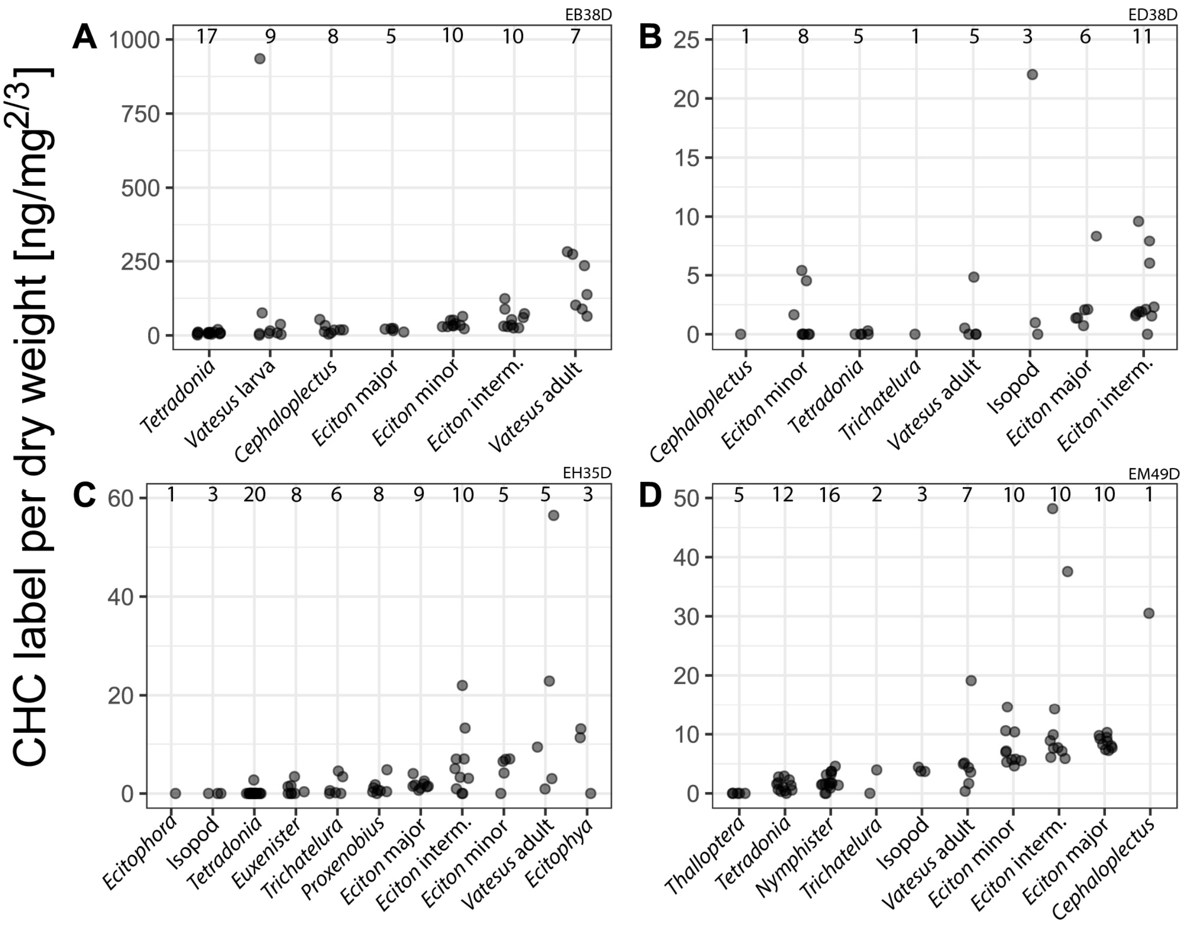

Figure S5. Shown are the concentrations of the label tetracosane-d50 detected on army ant workers and ecitophiles in one (A) *E. burchellii* colony (EB38D), (B) one *E. dulcium* colony (ED38D), one (C) *E. hamatum* colony (EH35D), and (D) one *E. mexicanum* colony (EM49D). For better data visualization species are lumped within their genera. Raw data in Additional file 1 include information at the species level. Sample sizes are given at the top of each subplot. Categories are ordered according to the groups' medians. Note the different scales on y-axes, suggesting that more label was initially transferred to workers in the *E. burchellii* colony compared to other colonies. Abbreviations: interm. = intermediate.

Supplemental references

1. Akino T. Chemical strategies to deal with ants: a review of mimicry, camouflage, propaganda, and phytomimesis by ants (Hymenoptera: Formicidae) and other arthropods. Myrmecol News. 2008;11:173–81.

2. Bagnères A-G, Lorenzi MC. Chemical deception/mimicry using cuticular hydrocarbons. In: Blomquist GJ, Bagnères A-G, editors. Insect hydrocarbons: biology, biochemistry, and chemical ecology. Cambridge: Cambridge University Press; 2010.

3. von Beeren C, Pohl S, Witte V. On the use of adaptive resemblance terms in chemical ecology. Psyche. 2012;https://doi.org/10.1155/2012/635761.

4. von Beeren C, Brückner A, Maruyama M, Burke G, Wieschollek J, Kronauer DJC. Chemical and behavioral integration of army ant-associated rove beetles–a comparison between specialists and generalists. Front Zool. 2018;15:8.

5. Witte V, Leingärtner A, Sabaß L, Hashim R, Foitzik S. Symbiont microcosm in an ant society and the diversity of interspecific interactions. Anim Behav. 2008;76:1477–86.

6. Polidori C, Geyer M, Schmitt T. Do *Sphecodes* cuckoo bees use chemical insignificance to invade the nests of their social *Lasioglossum* bee hosts? Apidologie. 2020;1–16.

7. Uboni A, Bagnères A-G, Christidès J-P, Lorenzi MC. Cleptoparasites, social parasites and a common host: chemical insignificance for visiting host nests, chemical mimicry for living in. J Insect Physiol. 2012;58:1259–64.

8. Cini A, Gioli L, Cervo R. A quantitative threshold for nest-mate recognition in a paper social wasp. Biol Lett. 2009;5:459–61.

9. Lenoir A, d'Ettorre P, Errard C, Hefetz A. Chemical ecology and social parasitism in ants. Annu Rev Entomol. 2001;46:573–99.

10. Martin SJ, Takahashi J, Ono M, Drijfhout FP. Is the social parasite *Vespa dybowskii* using chemical transparency to get her eggs accepted? Journal of Insect Physiology. 2008;54:700–7.

11. Lorenzi MC, d'Ettorre P. Nestmate recognition in social insects: What does it mean to be chemically insignificant? Frontiers in Ecology and Evolution. 2020;7:488.

12. Kühsel S, Brückner A, Schmelzle S, Heethoff M, Blüthgen N. Surface area–volume ratios in insects. Insect Sci. 2017;24:829–41.

13. Brückner A, Heethoff M, Blüthgen N. The relationship between epicuticular long-chained hydrocarbons and surface area-volume ratios in insects (Diptera, Hymenoptera, Lepidoptera). PLoS ONE. 2017;12:e0175001.

14. Schmelzle S, Heethoff M, Heuveline V, Lösel P, Becker J, Beckmann F, et al. The NOVA project: maximizing beam time efficiency through synergistic analyses of SRμCT data. Developments in X-Ray Tomography XI. International Society for Optics and Photonics; 2017. p. 103910P.

15. Crozier R, Dix MW. Analysis of two genetic models for the innate components of colony odor in social Hymenoptera. Behav Ecol Sociobiol. 1979;4:217–24.

16. von Beeren C, Schulz S, Hashim R, Witte V. Acquisition of chemical recognition cues facilitates integration into ant societies. BMC Ecol. 2011;11:30.

17. von Beeren C, Hashim R, Witte V. The social integration of a myrmecophilous spider does not depend exclusively on chemical mimicry. J Chem Ecol. 2012;38:262–71.

18. Rettenmeyer CW. Arthropods associated with Neotropical army ants with a review of the behavior of these ants (Arthropoda; Formicidae: Dorylinae). Lawrence: University of Kansas. PhD thesis; 1961.

19. Akre RD. Behavior of *Euxenister* and *Pulvinister* histerid beetles associated with army ants. Pan-Pac Entomol. 1968;44:87–101.
